# Refining an acute respiratory distress syndrome animal model supported by extracorporeal membrane oxygenator: incorporating clinically relevant mechanical ventilation strategy upon development

**DOI:** 10.1186/s40635-026-00892-7

**Published:** 2026-04-20

**Authors:** Keibun Liu, Gabriele Fior, Nchafatso Obonyo, Hideaki Nonaka, Angelo Milani, Gabriella Abbate, Sofia Portatadino, Chiara Palmieri, Shinichi Ijuin, Kei Sato, Silver Heinsar, Sun Kyun Ro, Lucia Gandini, Kota Hoshino, Noriko Sato, Samia Farah, Carmen Ainola, Margaret R. Passmore, Rachana Panduru, Mahe Bouquet, Emily S. Wilson, Kieran Hyslop, Molly-rose McInerney, Cheng Zhang, Caitlin McGrath, Joshua Paech, Jacky Y. Suen, John F. Fraser, Gianluigi Li Bassi

**Affiliations:** 1 Critical Care Research Group, The Prince Charles Hospital, Brisbane, Australia; 2 Institute for Molecular Bioscience, The University of Queensland, Brisbane, Australia; 3 Non-Profit Organization ICU Collaboration Network (ICON), Tokyo, Japan; 4 School of Veterinary Science, The University of Queensland, Gatton, Australia; 5 Department of Cardiology, North Estonia Medical Centre, Tallinn, Estonia; 6 Institute of Clinical Medicine, University of Tartu, Tartu, Estonia; 7 Faculty of Medicine, The University of Queensland, Brisbane, Australia; 8 St. Andrews War Memorial Hospital, Brisbane, Australia; 9 School of Biomedical Sciences, Faculty of Medicine, University of Queensland, Brisbane, Australia; 10 School of Pharmacy and Medical Sciences, Griffith University, Southport, Australia; 11 Queensland University of Technology, Brisbane, Australia; 12 Intensive Care Unit, The Prince Charles Hospital, Chermside, Australia

**Keywords:** Acute respiratory distress syndrome, Acute lung injury, Extracorporeal membrane oxygenation, Lipopolysaccharide, Mechanical ventilation, Positive end-expiratory pressure, Oleic acid

## Abstract

**Background:**

Acute respiratory distress syndrome (ARDS) requiring veno-venous extracorporeal membrane oxygenation (VV-ECMO) is associated with high mortality. One of the primary challenges in translating preclinical findings into effective clinical treatments lies in developing animal models that accurately replicate clinical scenarios. Thus, we aimed to develop a clinically relevant novel ovine model of severe ARDS with VV-ECMO support, with the primary aim of assessing feasibility through 48-h survival, while monitoring safety and clinical relevance.

**Methods:**

Six sheep (52.7 ± 1.5 kg) were anesthetized and mechanically ventilated. Severe ARDS was induced by oleic acid and lipopolysaccharide (0.5 µg/kg). Lung-protective mechanical ventilation commenced once the PaO_2_/FiO_2_ ratio deteriorated to less than 150 mmHg, with additional doses of oleic acid administered if the PaO_2_/FiO_2_ improved. Severe ARDS criteria, triggering ECMO initiation, were defined as PaO_2_/FiO_2_ < 100 mmHg, PaCO_2_ > 60 mmHg, or refractory respiratory acidosis (T0) before commencing VV-ECMO (T1), followed by a 48-h observation. The primary outcome was survival at 48 h to assess the feasibility of a novel model. All complications were also recorded, and lung tissues were obtained upon autopsy. Assessments followed the American Thoracic Society (ATS) Workshop Report 2022 recommendations, including histological assessments, alveolar–capillary barrier evaluations, and inflammatory and physiological responses. Data were analysed using the Friedman test.

**Results:**

All sheep survived the 48-h follow-up. No complications were recorded throughout the study. In all sheep, although PaO_2_/FiO_2_ reached 126 (interquartile range: IQR 103–149) mmHg, lung-protective mechanical ventilation strategies improved PaO_2_/FiO_2_ to 181 (IQR: 167–185) mmHg within 60 min, requiring additional oleic acid doses to reach injury criteria. All sheep developed hallmark features of experimental ARDS including histological evidence (filling of the alveolar space with proteinaceous alveolar fluid and debris and increasing histologic injury score), impaired alveolar–capillary barrier (elevated total protein in bronchoalveolar lavage fluid (BAL) and increased lung wet-to-dry weight ratio), inflammatory response (increase in IL6, IL-8 and neutrophil numbers in BAL), and physiologic dysfunction (e.g., impaired oxygenation, reduction in lung compliance).

**Conclusions:**

We developed a novel animal model of ARDS that closely replicates ARDS management, including lung-protective mechanical ventilation before the initiation of VV-ECMO, ensuring a prolonged 48-h survival observation without any complications. This model meets all four key features of ARDS as recommended by the latest ATS guidelines and provides an innovative platform to support clinical translation.

**Supplementary Information:**

The online version contains supplementary material available at 10.1186/s40635-026-00892-7.

## Introduction

Despite extensive research over the past 50 years, clinical management of acute respiratory distress syndrome (ARDS) is only supportive, with extremely high mortality in the most severe cases[1, 2]. Current clinical practice guidelines recommend lung-protective ventilation, prone positioning, neuromuscular blockades, and veno-venous extra-corporeal membrane oxygenation (VV-ECMO) for severe ARDS[3–5]. VV-ECMO has a critical role as a bridge to lung recovery. Recent evidence from the SARS-CoV-2 pandemic has further confirmed the benefits of using VV-ECMO[6]. However, over 30% of patients with severe ARDS still died despite the best management even in the recent randomized controlled trial [7].

In this context, preclinical studies have a crucial role in investigating, testing, and revolutionizing the management of ARDS[8]. Large animal models have been employed to evaluate the efficacy and safety of novel interventions and equipment, prior to translation into clinical trials, owing to their similarities in body size, anatomy, and respiratory physiology[9]. Many treatments were tested in preclinical settings prior to translation into clinical, i.e. lung-protective ventilation with low tidal volume [10], beta-2 agonist [11], recruitment maneuvers [12], VV-ECMO[13]. However, some of the interventions tested ultimately failed to translate into clinical settings, potentially stemming from limitations in the design and protocols of the preclinical studies[8, 14]. Our recent systematic review highlighted that existing preclinical models of ARDS supported by VV-ECMO lack clinical relevance in terms of the severity of injury, criteria for VV-ECMO initiation, and overall management, raising concerns about translational potential[15]. Moreover, to the best of our knowledge, existing animal models do not incorporate high levels of PEEP or other lung-protective ventilation strategies prior to initiating VV-ECMO [15]. For instance, in clinical settings, PEEP, FIO₂, tidal volume, and driving and plateau pressures are carefully titrated based on pulmonary function improvements; however, such an approach has rarely been applied in preclinical models. This may lead to an underestimation of lung injury severity compared to clinical settings[16], potentially causing discrepancies in the observed responses to treatments that could otherwise demonstrate meaningful effects in clinical practice.

The American Thoracic Society (ATS) has recently updated recommendations for experimental models of ARDS, which comprise four key domains to increase the likelihood of translating preclinical findings and to reflect the key pathophysiologic features and underlying biology of human ARDS[14]. The scoping review investigating the current preclinical models of ARDS also highlighted that the existing models have not met the standards outlined by the ATS[15], indicating the limited translatability of the current preclinical models and the urgent need to bridge this gap[1].

Therefore, we sought to develop a novel ovine model of severe ARDS with a consistent degree of respiratory impairment that could closely mirror clinical indications for VV-ECMO. The primary objective of our study was to assess the model’s feasibility by survival up to 48 h after the initiation of VV-ECMO, while comprehensively monitoring complications for safety and key clinical variables for translatability.

## Methods

**Fig. 1:**
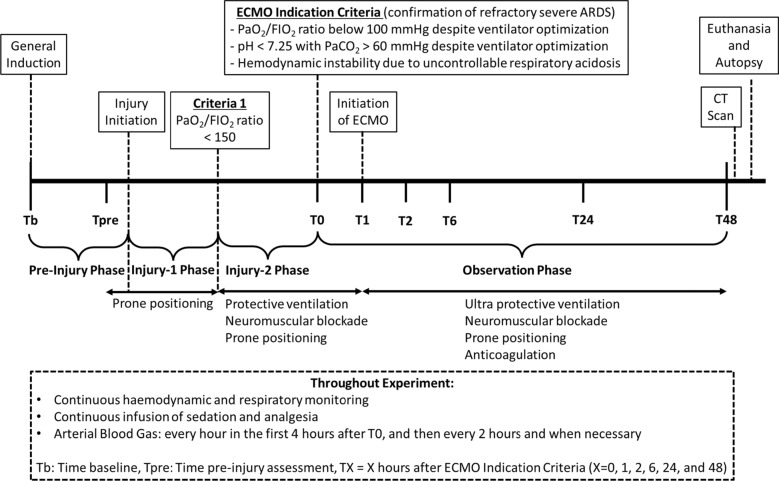
Experimental timeline. ARDS: acute respiratory distress syndrome, CT: computed tomography, ECMO: extracorporeal membrane oxygenation, FIO_2_: fraction of inspired oxygen, PaCO_2_: partial pressure of carbon dioxide, PaO_2_: partial pressure of oxygen

Details of the study settings are included in Additional file 1: Table S1. Briefly, all sheep (aged between 1 and 3 years old and weighing between 40 and 70 kg) passed a comprehensive and satisfactory pre-operative health assessment (e.g., physiological assessments such as infection and full blood count), and were fasted overnight with free access to water before experimentation. The experimental timeline comprised *Pre-Injury*, *Injury-1st and -2nd*, and *Observation Phases* (Fig. 1). The study was conducted in accordance with the ARRIVE guidelines.

### Pre-injury phase

All the procedures listed in Table 1 were performed under sterile conditions. After endotracheal intubation, mechanical ventilation commenced (Galileo 5, Hamilton Medical). General anaesthesia was achieved with ketamine (2.5–15 mg/kg/h), midazolam (0.5–3.0 mg/kg/hr), and fentanyl (5–40 mcg/kg/h). Following a fluid challenge with 250 ml of Hartmann’s solution, maintenance fluid (1ml/kg/h) commenced. Mechanical ventilation protocol was applied to modulate ARDS clinical recommendations (Additional file 1: Fig. S1) [4, 5, 17]. The animal was placed in the prone position and haemodynamic, respiratory monitoring, and clinical management were applied as reported in Table 2 and Additional file 1: Fig. S2.

**Table 1:** The list of procedures during Pre-Injury Phase

Order	Procedure	Site	Size	Comments
In a prone position on a custom-made sling				
1	Central venous line	Left external jugular vein (proximal)	Quad lumens	For general anaesthesia, fluid bolus, and other drug injections
2	Swan–Ganz sheath	Left external jugular vein (distal)	8.5–9 Fr	
3	Endotracheal intubation	Through mouth	9 Fr	Before the intubation, prophylactic doses of cefazolin (1 g) and gentamicin (80 mg) were administered. Intubation was performed following injections of 1 mg/Kg of midazolam and propofol 3–4 mg/Kg
In a supine position on the surgical table				
4	Urinary catheter insertion		12 Fr	
5	Orogastric tube insertion		10–12 Fr	This was a non-sterile procedure. The mouth was opened by laryngoscope and the tube was advanced by Magiru Forceps if necessary
5	Central venous line	Left femoral venous	Quad lumens	Only for the use of catecholamines
6	Arterial line	Left femoral artery	6 Fr	For arterial pressure monitoring, blood gas, and blood samplings
7	Introducer sheath	Right femoral and external jugular vein	6–8 Fr	For ECMO cannulation
8	Swan–Ganz catheter	Through the sheath on the left neck	8.5 Fr (Edwards)	Calibration was performed by placing the transducer at the level of the heart and verifying transducer zeroing. Mixed venous blood gas was used for calibration of the Vigilance II monitoring system
9	Tracheostomy		10 Fr (Softseal Portex)	The endotracheal tube was removed after the completion
10	Chest drainage tubes	Both thoracic cavity	28–32 Fr (Argyle thoracic catheter)	Prevention for tension pneumothorax. The tubes were connected to Atrium Oasis Dry Suction Water Seal Chest Drain system
10.5	Before ECMO cannulations, 60 units/kg of unfractionated heparin was injected			
11	ECMO drainage cannula	Right external jugular vein	25 Fr, 38 cm (HLS, Getinge)	The pre-inserted sheath was removed. This was performed under fluoroscopy. The tip was placed 1–2 cm deeper from the junction of the supra venous cava and right atrium
12	ECMO return cannula	Right femoral vein	17 Fr, 23 cm (HLS, Getinge)	The pre-inserted sheath was removed. This was performed under fluoroscopy
	The two inserted cannulas were locked with heparinized saline (2000 units of unfractionated heparin in 1000 ml of 0.9% saline) until connected to the ECMO circuit at T1			
13	One hour of rest for stabilization in a prone position until the initiation of injuries

**Table 2:** Standard monitoring settings

Monitoring	Device	Parameter to monitor
Standard haemodynamic monitoring		
Physiological monitor	CARESCAPE B650 monitor (GE Health Care)	ECG waveforms, HR, SBP, DBP, MAP, CVP, SPAP, DPAP, MPAP, EtCO_2_, and SpO_2_ were continuously monitored
Swan–Ganz monitor	Vigilance II (Edwards Lifesciences)	SvO_2_, CO, and temperature were continuously monitored
Cardiac function monitoring	a Vivid-i ultrasound machine (GE Vingmed Ultrasound)	Ultrasound assessments of cardiac systolic and diastolic functions were performed at Tb, Tpre, T0, T2, T6, and every 6 h until T48 Left ventricular filling pressure (PCWP) was monitored at least hourly and when necessary (See Table 3 for the details of cardiac function measurements)
Respiratory monitoring		
Respiratory mechanics on the ventilator	HAMILTON-G5 (Hamilton Medical)	TV, RR, MV, PIP, PEEP, Plateau pressure, and Static compliance were continuously monitored on the ventilator monitor
Calculation of respiratory mechanics		Driving pressure: Plateau pressure—PEEP Dead space ventilation: (PaCO_2_—EtCO_2_)/PaCO_2_
General monitoring for in–out fluid balance		
Fluid administration		Hourly amount of total fluid infusion to calculate fluid input
Urinary output		Hourly amount of urine to calculate fluid output
Pleural effusion monitor	Atrium Oasis Dry Suction Water Seal Chest Drain, 2000 ml (Getinge)	Hourly amount of pleural effusion to calculate fluid output
Orogastric fluid		Hourly amount of orogastric fluid to calculate fluid output
In–out fluid balance		(Total input)–(total output)

CO: cardiac output, CVP: central venous pressure, DBP: diastolic blood pressure, DPAP, diastolic pulmonary arterial pressure, ECG: electrical cardiogram, EtCO_2_: end-tidal carbon dioxide, HR: heart rate, MAP, mean arterial pressure, MPAP: mean pulmonary arterial pressure, MV: minute ventilation, PCWP: pulmonary capillary wedge pressure, PEEP: positive end-expiratory pressure, PIP: peak inspiratory pressure, RR: respiratory rate, SBP: systolic blood pressure, SPAP: systolic pulmonary arterial pressure, SpO_2_: percutaneous oxygen saturation, SvO_2_: venous oxygen saturation (in the pulmonary artery), Tb: time baseline, Tpre: time pre-injury assessment, TX = X hours after ECMO Indication Criteria (X = 0, 1, 2, 6, 24, and 48)

### Injury-1st phase: development of moderate ARDS

Oleic Acid (OA: 0.3 ml/kg per one-time) was administered through the proximal port of the Swan–Ganz catheter over 2 min. When mean arterial blood pressure (MAP) decreased below 65 mmHg during or after OA administration, intravenous metaraminol boluses (1–2 mg) were administered. If hypotension was refractory to metaraminol boluses, continuous infusion of noradrenaline was initiated as per protocol (Additional file 1: Fig. S2). Two assessments of PaO_2_/FIO_2_ ratio at 15 and 30 min after OA administration were obtained via arterial blood gases (ABG, ABL-90 FLEX analyzer, Radiometer Medical ApS, Brønshøj, Denmark) (Additional file 1: Fig. S3). Mechanical ventilation settings were not altered in this phase, except for the respiratory rate (RR) that was titrated to avoid life-threatening respiratory acidosis (Additional file 1: Fig. S1). ***Injury Criterion-1*** required achieving moderate-to-severe ARDS, defined as a PaO2/FiO2 ratio of < 150 mmHg, in at least one of two ABG measurements. OA injection of the one-time dose of 0.3 ml/kg was repeated if the criterion was not met in the two assessments of oxygenation.

### Injury-2nd phase: development of refractory severe ARDS

Following achievement of criterion 1, lung-protective ventilation commenced (Additional file 1: Fig.S1), including high levels of PEEP and neuromuscular blockade (vecuronium: 2 mg/h). Then, continuous infusion of lipopolysaccharide (LPS: 0.05 μl/kg body weight in 50 ml of saline) was administered intravenously over one hour, and ABGs were obtained at 15, 30, 45, and 60 min. Of note, additional OA infusions (0.3 ml/kg) were administered in case lung-protective ventilation improved PaO_2_/FiO_2_. ***VV-ECMO Indication Criterion-2*** required achieving refractory severe ARDS, defined by PaO_2_/FIO_2_ < 100, pH < 7.25 and PaCO_2_ > 60 mmHg, or severe haemodynamic instability associated with respiratory acidosis (Additional file 1: Fig. S3 and S4) with no response to lung-protective ventilation adjustments [7].

### Observation phase

Upon ***VV-ECMO Indication Criterion-2*** (T0), animals were monitored for 48 h. At T1, the pre-inserted ECMO cannulas were connected to the circuit (SOLAS circuit, MERA, Tokyo, Japan). Details of ECMO support are in Additional file 1: Fig. S5 [7, 17–19]. After the initiation of ECMO support and throughout the follow-up period, ultra-protective ventilation was applied (Additional file 1: Fig. S6). Management of coagulation and electrolytes is shown in Additional file 1: Table S2. Blood, bronchoalveolar lavage fluid (BAL), ultrasound, and organ tissue were utilized to monitor the severity of ARDS injury (Additional file 1: Fig. S7).

### Outcomes

The primary outcome was survival at 48h. Any possible complications related to ECMO (i.e. bleeding and thrombosis) (Additional file 1: Table S3) were recorded. The model was appraised against the experimental ARDS criteria outlined in the latest ATS Workshop Report to ensure alignment with established recommendations (Table 3) [14]. Ten fields were analysed by one non-blinded experienced pathologist for the histological assessments of the lung tissue. The lung wet-to-dry ratios were compared with the reference in a previously published ECMO model without any injuries [20].

**Table 3:** Features to assess the suitability of an experimental ARDS animal model

Feature	Variables to be evaluated
Features and measurements of experimental ARDS	
Histological evidence of tissue injury	Alveolar space (proteinaceous alveolar fluid, debris, fibrin, neutrophil infiltration, hyaline membranes) Lung Injury Score (a validated histologic injury score) Alveolar epithelial injury Alveolar septum and/or interstitial space Damage pattern
Alteration of the alveolar–capillary barrier	Lung wet-to-dry ratio Total protein in bronchoalveolar lavage fluid,
Presence of an inflammatory response	IL-6 and neutrophil numbers in bronchoalveolar lavage fluid
Evidence of physiological dysfunction	Oxygenation in arterial blood gas (PaO_2_) Non-invasive measurements of oxygenation: SpO_2_ Respiratory mechanics (compliance, plateau pressure, driving pressure, tidal volume)
Excluding other potential causes of ARDS	
Cardiac function	Systolic cardiac function (left ventricular ejection fraction: LVEF) and diastolic cardiac function (end-diastolic strain rate: EDSR), which were measured by ultrasound, and left ventricular filling pressure, which was measured by Swan–Ganz catheter (pulmonary capillary wedge pressure: PCWP)

ARDS: acute respiratory distress syndrome,

Cardiac function was assessed using echocardiography (GE Vingmed Ultrasound, Horten, Norway) with a 3Sc probe at Tecmo. Measurements were taken at Tb, Tpre, T0, T2, T6, and every 6 h until T48. In sheep, transthoracic echocardiography does not provide an apical longitudinal view as in humans due to anatomical constraints; therefore, alternative two- and four-chamber longitudinal views were obtained from the anterior and lateral positions of the LV wall, respectively. Based on these views, LV ejection fraction was calculated using Simpson’s method. Diastolic function was further assessed using strain analysis from short-axis views at three levels: basal, mid, and apical. Early peak circumferential diastolic strain rates were measured at each level and averaged for diastolic function analysis [21]. All echocardiography analysis was conducted by an experienced cardiologist using the TomTec-Arena analysis platform (TomTec Imaging Systems GMBH, Unterschleissheim, Germany)

### Statistical analysis

Continuous variables were presented as median and interquartile range (IQR) or median with 95% confidence interval (CI). Longitudinal data were analysed using the Friedman test to assess overall time-dependent differences. When the Friedman test yielded significant results, post hoc comparisons among times of assessments were performed using the Wilcoxon signed-rank test. Bonferroni correction was applied to adjust for multiple comparisons. Two-sided adjusted *p*-values < 0.05 were considered statistically significant. The lung wet-to-dry ratio was compared by t-test to the reference of a published ECMO model without injury, which was described with the mean and the standard error of the mean in the results [20]. All analyses were performed using GraphPad Prism version 10.0.

## Results

### ***Baseline characteristics (***Table 4***)***

**Table 4:** Baseline characteristics

Variable	Overall (n = 6)
Height, cm	110 [107.3–112.5]
Weight, kg	52.5 [49.5–56.3]
**Physiological hasemodynamic parameter**	
Heart rate, beats/min	79.0 [65.5–101]
Systolic blood pressure, mmHg	126.5 [114.5–147.0]
Diastolic blood pressure, mmHg	88.0 [67.8–91.0]
Mean arterial pressure, mmHg	107.5 [98.0–120.0]
Central venous pressure, mmHg	1.5 [0.3–2.0]
Systolic pulmonary artery pressure, mmHg	16.0 [16.0–18.0]
Diastolic pulmonary artery pressure, mmHg	9.5 [7.5–10.0]
Mean pulmonary artery pressure, mm Hg	12.5 [11.3–14]
EtCO_2_, mmHg	42.5 [40.5–43.0]
SpO_2_, %	95.5 [92.8–97.0]
Mixed venous oxygen saturation, %	81.5 [71.0–83.0]
Cardiac output, L/min	5.9 [5.3–6.7]
Temperature, °C	37.6 [36.6–37.9]
**Respiratory function and mechanics**	
Plateau pressure, cmH_2_O	16.1 [13.5–16.9]
Driving pressure, cmH_2_O	11.1 [8.5–11.9]
Static compliance, ml/cmH_2_O	35.9 [34.3–36.6]
Dead space ventilation, %	8.0 [4.6–12.2]
pH	7.40 [7.38–7.41]
PaCO_2_, mmHg	46.8 [45.2–49.1]
PaO_2_/FIO_2_ ratio, mmHg	540.3 [530.6–571.4]
**Cardiac function**	
Systolic cardiac function: LVEF, %	57.1 [56.3–57.3]
Diastolic cardiac function: EDSR	1.10 [1.06–1.17]
Left ventricular filling pressure: PCWP, mm Hg	5.0 [5.0–6.0]
**Haematological and biochemical parameter**	
Na, mmol/L	141.5 [140.3–142]
K, mmol/L	3.9 [3.8–3.9]
Ca, mmol/L	1.2 [1.1–1.2]
Cl, mmol/L	103.5 [102.3–106.0]
Glucose, mmol/L	3.6 [3.2–4.1]
Lactate, mmol/L	0.6 [0.4–0.9]
Base excess, mmol/L	4.0 [2.5–5.5]
Bicarbonate, mmol/L	28.2 [26.5–28.8]

Data in table are shown as a median with interquartile range

FIO_2_: fraction of inspired oxygen, EDSR: end-diastolic strain rate, LVEF: left ventricular ejection fraction, PaCO_2_: partial pressure of carbon dioxide, PaO_2_: partial pressure of oxygen, PCWP: pulmonary capillary wedge pressure

Baseline characteristics of six sheep are shown in Table 4. Physiological haemodynamic parameters, respiratory function and mechanics, cardiac function, and haematological and biochemical parameters were within the normal range.

### Characteristics of the injury phases

#### Injury-1st phase

**Fig. 2:**
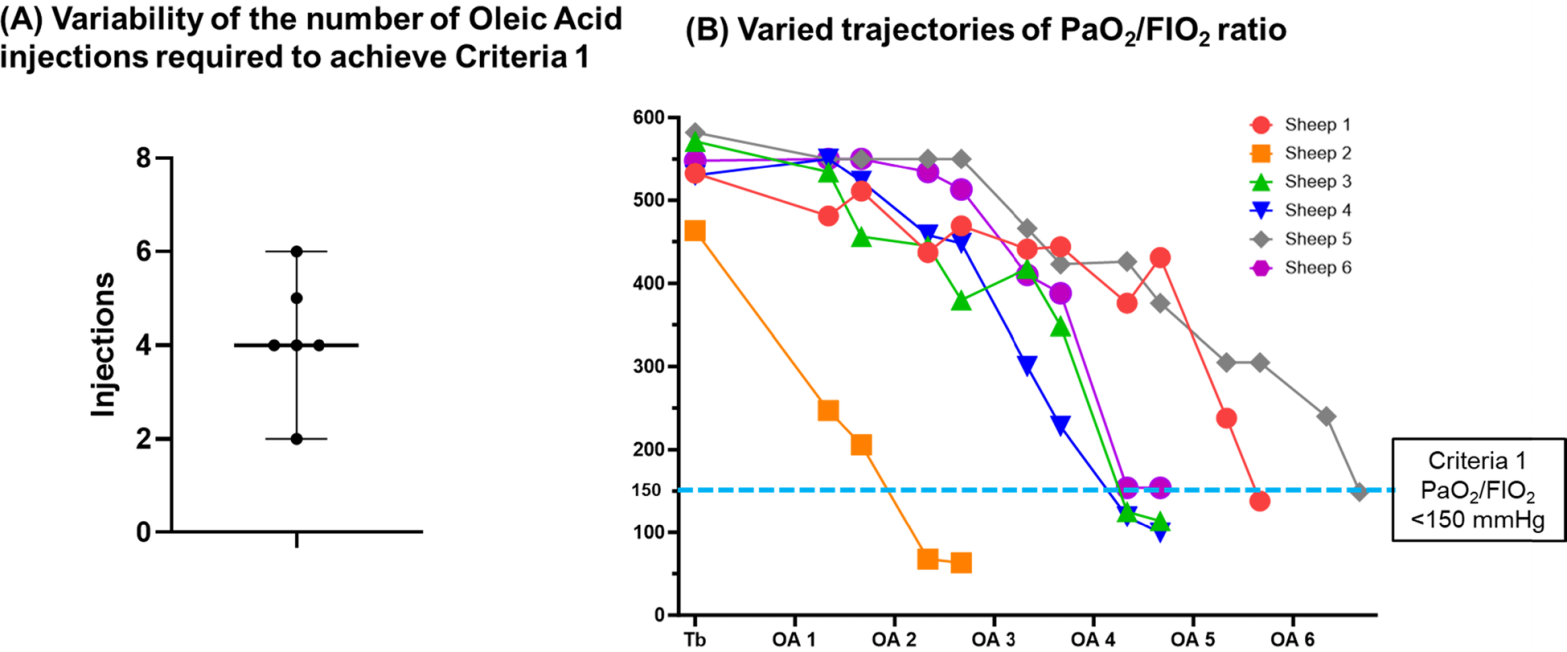
Injury characteristics in Injury Phase 1 under fixed ventilator settings. **A** Variability of the number of oleic acid injections required to achieve Criteria 1. **B** Varied trajectories of PaO_2_/FIO_2_ ratio. FIO_2_: fraction of inspired oxygen, PaO_2_: partial pressure of oxygen

***Criterion-1*** was achieved with a variable number of OA administrations, ranging from 2 to 6 (Fig. 2A). A downward trend of PaO_2_/FIO_2_ over the Injury-1st Phase was found (Fig. 2B), but the PaO_2_/FIO_2_ impairment response after OA administrations varied across animals. Upon achievement of ***Criterion-1***, the mean PaO_2_/FIO_2_ ratio was 119.6 (± 14.2) mmHg.

#### Injury-2nd phase

**Fig. 3:**
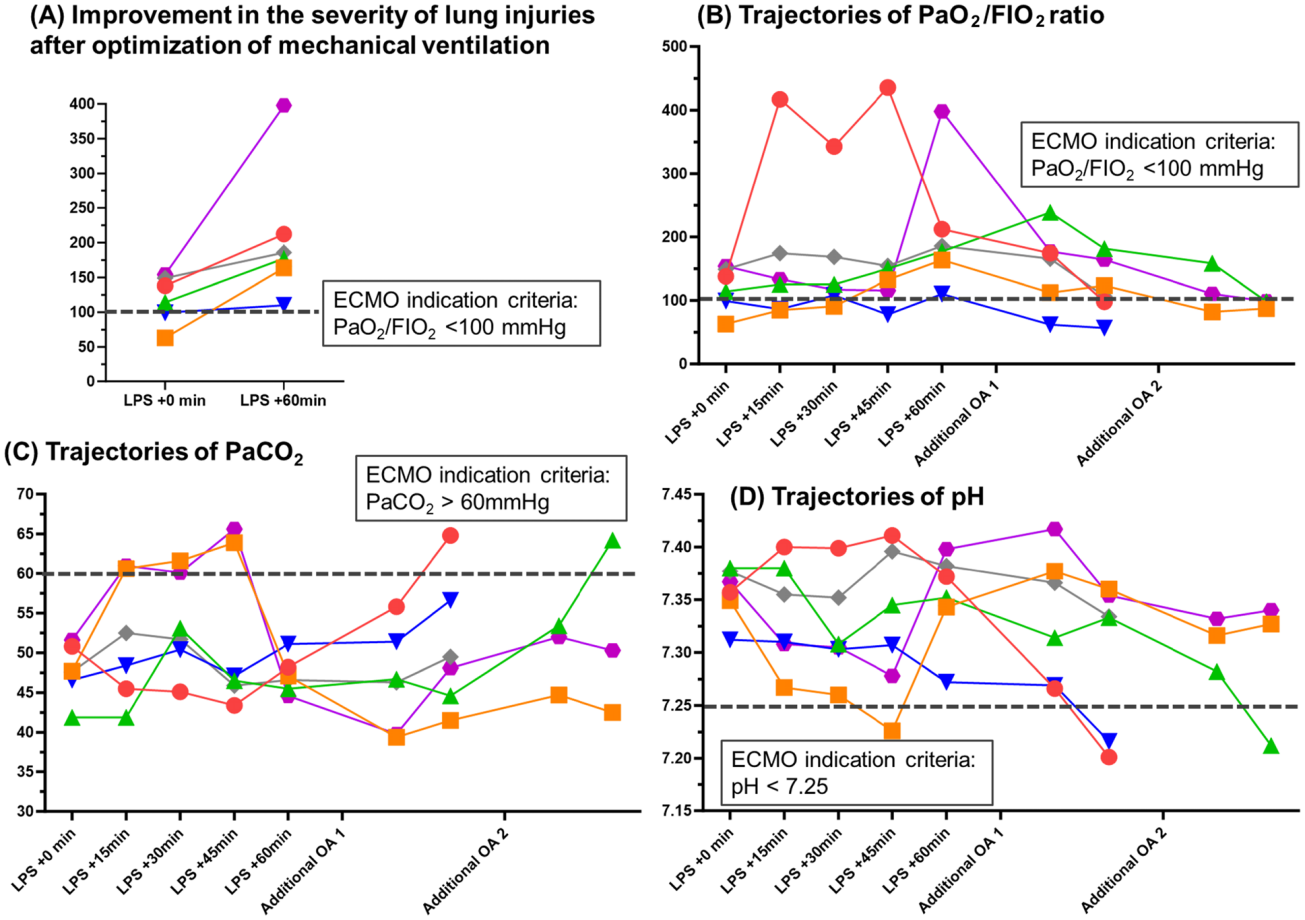
Varied injury levels and trajectory in Injury Phase 2 after an aggressive mechanical ventilator strategy. **A** Improvement in the severity of lung injuries after optimization of mechanical ventilation. **B** Trajectories of PaO_2_/FIO_2_ ratio. **C** Trajectories of PaCO_2_. **D** Trajectories of pH. FIO_2_: fraction of inspired oxygen, PaCO_2_: partial pressure of carbon dioxide, PaO_2_: partial pressure of oxygen

**Fig. 4:**
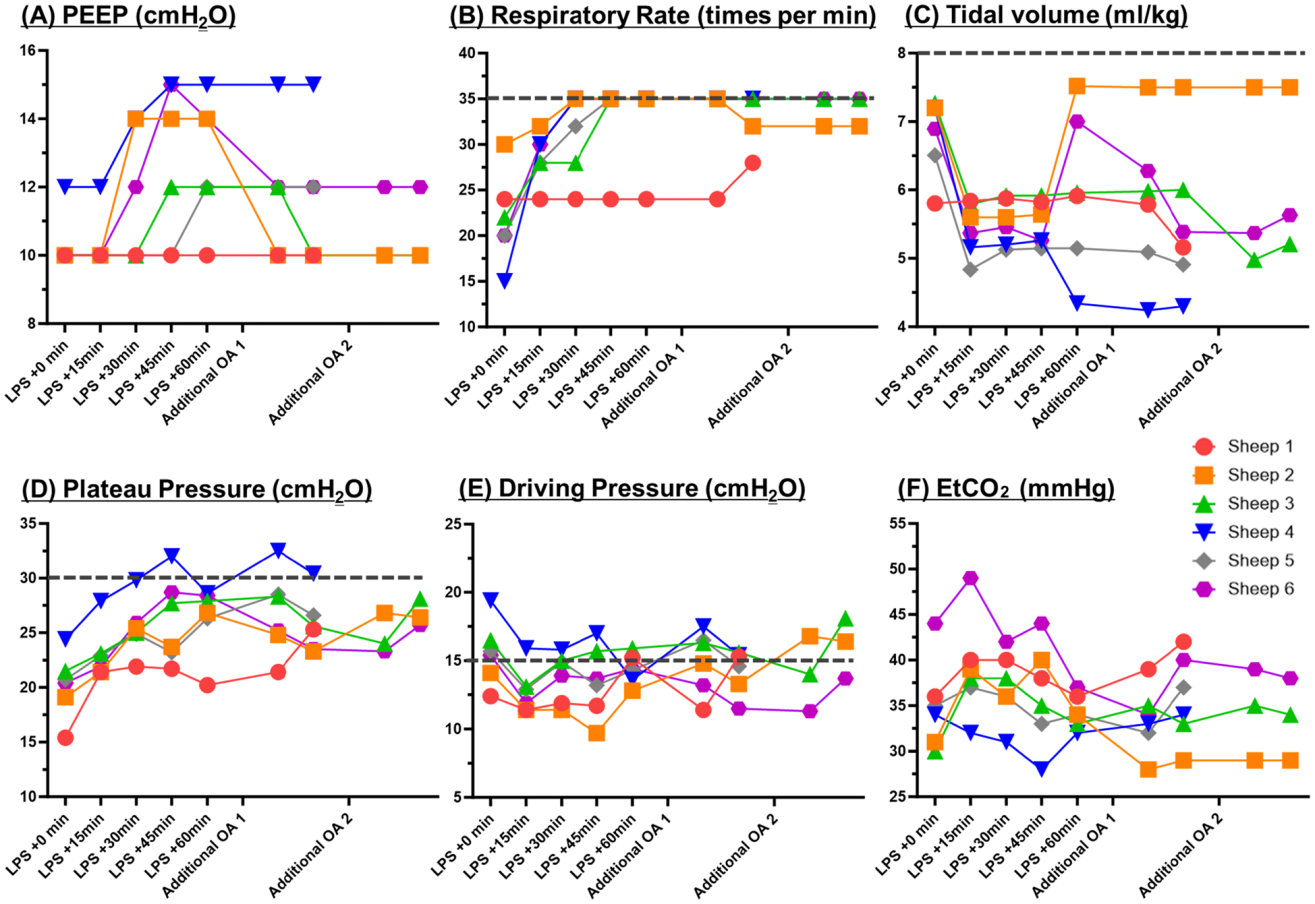
Optimization of mechanical ventilator settings in Injury Phase 2. EtCO_2_: end-tidal carbon dioxide, LPS: lipopolysaccharide, OA: oleic acid, PEEP: positive end-expiratory pressure

One hour after OA infusion, lung-protective ventilation and neuromuscular blockade significantly improved the PaO_2_/FIO_2_ ratio from 119.6 (± 14.2) to 207.8 (± 40.5) mmHg (Fig. 3A). As oxygenation and gas exchange function deteriorated, PEEP was increased to 13 (± 0.8), RR to 34 (± 1.1), and tidal volume was increased from 4.3 to 7.5 ml/kg (Fig. 4). The plateau pressure and driving pressure were monitored and kept consistently below 30 and 15 cmH_2_O, respectively (Fig. 4). After the completion of LPS infusion, one or two additional OA injections were required for all sheep to achieve ***VV-ECMO Indication Criteria***. All sheep met ***VV-ECMO Indication Criterion*** (PaO_2_/FIO_2_ < 100 mmHg) (Fig. 3B). Two met the ***VV-ECMO Indication Criterion*** of hypercapnia, despite lung-protective ventilation (Fig. 3C and D). Severe haemodynamic instability due to respiratory acidosis was not observed in any sheep. Upon reaching the ***VV-ECMO Indication criterion,*** the mean PaO_2_/FIO_2_ ratio, PaCO_2_, and pH were 91.0 (± 7.3) mmHg, 54.7 (± 3.6) mmHg, and 7.27 (± 0.03), respectively.

### Outcomes

**Fig. 5:**
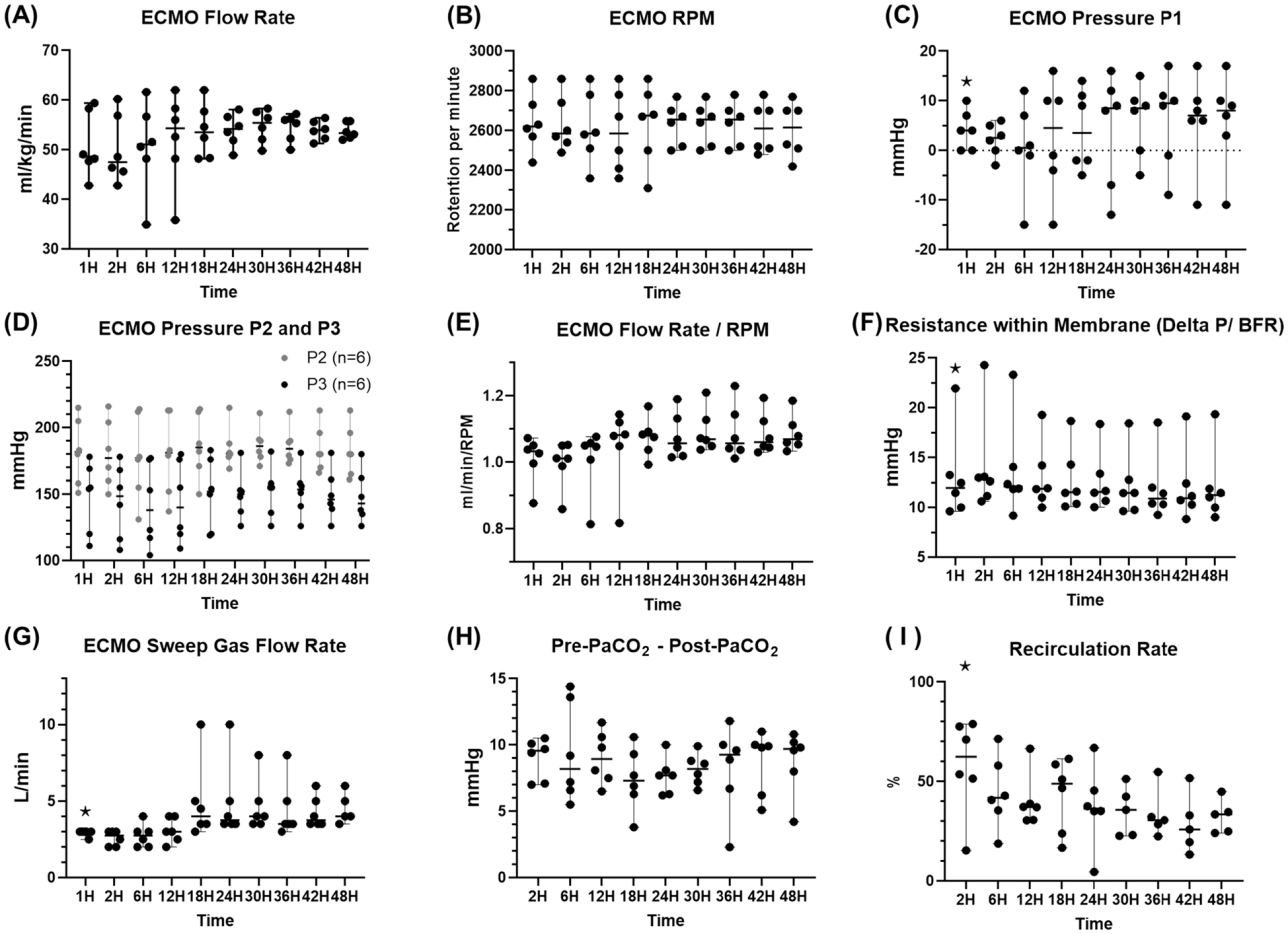
ECMO performances in the observation phase. ⋆: *p* value < 0.05 with the Friedman test. BFR: blood flow rate of ECMO, Delta P: P2-P3, ECMO: extracorporeal membrane oxygenation, Pre-PaCO_2_: partial pressure of carbon dioxide in the pre-oxygenator blood, Post-PaCO_2_: partial pressure of carbon dioxide in the post-oxygenator blood, RPM: rotation per minute

**Fig. 6:**
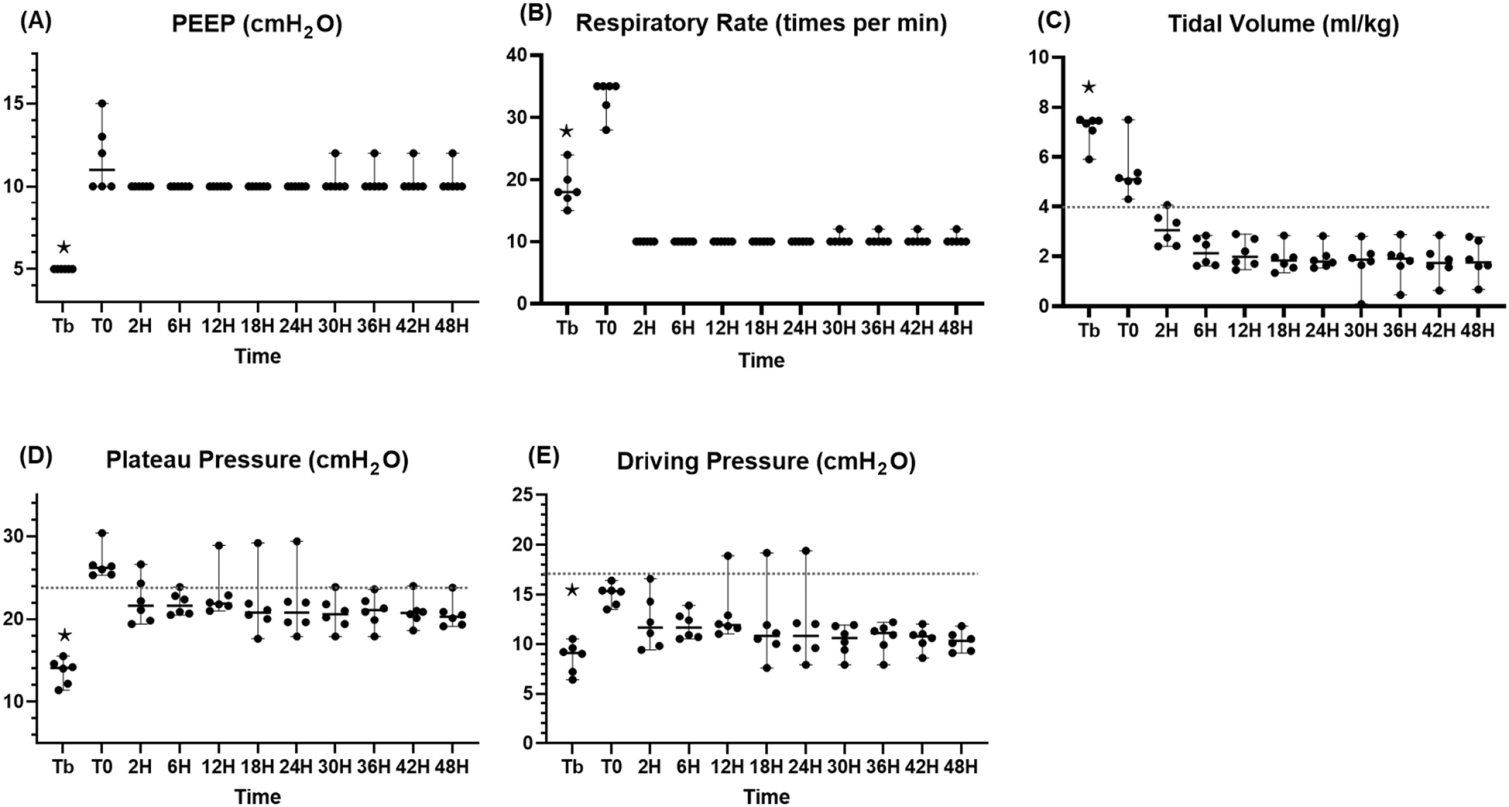
Lung-protective strategy in the observation phase. **A** PEEP (mmHg), **B** respiratory rate (%), **C** tidal volume (ml/kg), **E** plateau pressure, **F** driving pressure. ⋆: *p* value < 0.05 with the Friedman test. PEEP: positive end-expiratory pressure

All six sheep survived until the end of the experiment (T48) and completed all pre-mortem and post-mortem assessments. Adverse events or complications were not observed in any sheep. This was also confirmed by brain CT scanning, corroborating the absence of brain bleeding or foci of ischaemic changes in all sheep. Throughout the 48-h observation period, VV-ECMO flow rate (a mean of 2.7 ± 0.1 L/min), rotation per minute, venous access, and pre- and post-oxygenator pressures, and resistance within the oxygenator (Fig. 5A–F) remained stable. The sweep gas flow rate was kept at 4.0 (± 0.1) L/min, and oxygenator function parameters (Pre-oxygenator PaCO_2_ – Post-oxygenator PaCO_2_) were consistent throughout the experiments (Fig. 5G and H). Recirculation rate started at 58.0 ± 9.8% at T2, followed by a consistent reduction up to 32.4 ± 3.8% at T48 (Fig. 5I). The ECMO support, with the mechanical ventilation settings of FIO_2_ (40%), PEEP (10 cmH_2_O), RR (10 times/min), and tidal volume (from 1.7 to 3.9 ml/kg), consistently achieved the planned targets for lung-protective ventilation of plateau and driving pressure throughout the observation period (Fig. 6).

### Lung injury

#### Histology

**Fig. 7:**
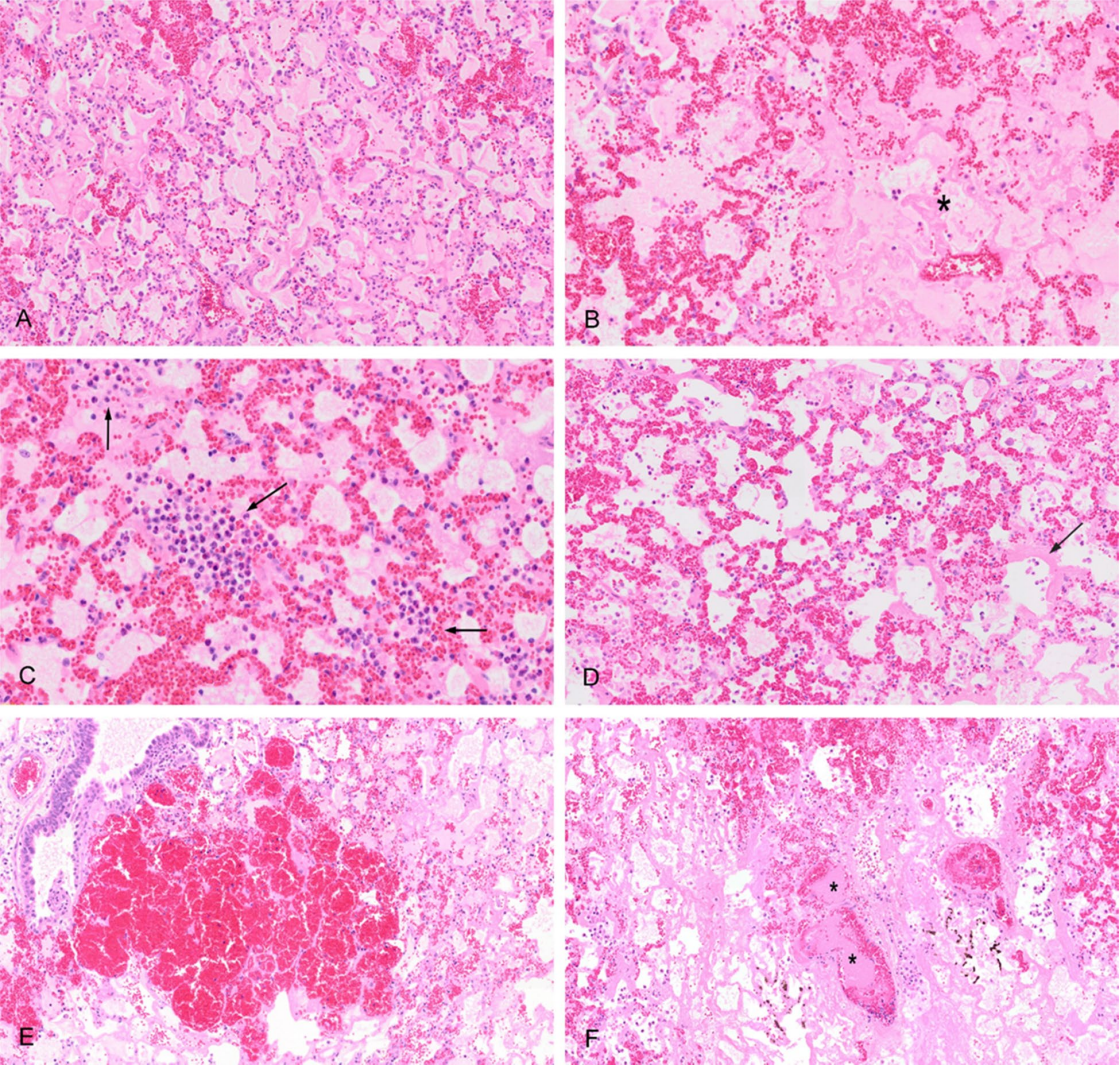
Histological evidence. Representative haematoxylin–eosin stained tissue sections demonstrating the main pulmonary histological lesions. **A** Accumulation of proteinaceous material within the alveoli. **B** Focal area of alveolar injury (asterisk) with severe degenerative changes of the endothelial, epithelial, and septal components. **C** Intraalveolar aggregates of high numbers of neutrophils (arrows). **D** Diffuse thickening of the alveolar septa due to congestion (dilation of alveolar capillaries). Note the hyaline membrane (arrow) suggestive of alveolar damage. **E** Intraalveolar haemorrhages. **F** Multiple thrombi (asterisks) surrounded by areas of necrosis and haemorrhage

As shown in Fig. 7, the following features were observed in all the animals: accumulation of proteinaceous debris (either fibrin or oedematous material) within the alveolar space, presence of neutrophils within the alveoli and in the alveolar septa, thickening of the alveolar septa, due to congestion and/or oedema. None of the samples showed the formation of hyaline membranes.

#### Alveolar–capillary barrier disruption

**Fig. 8:**
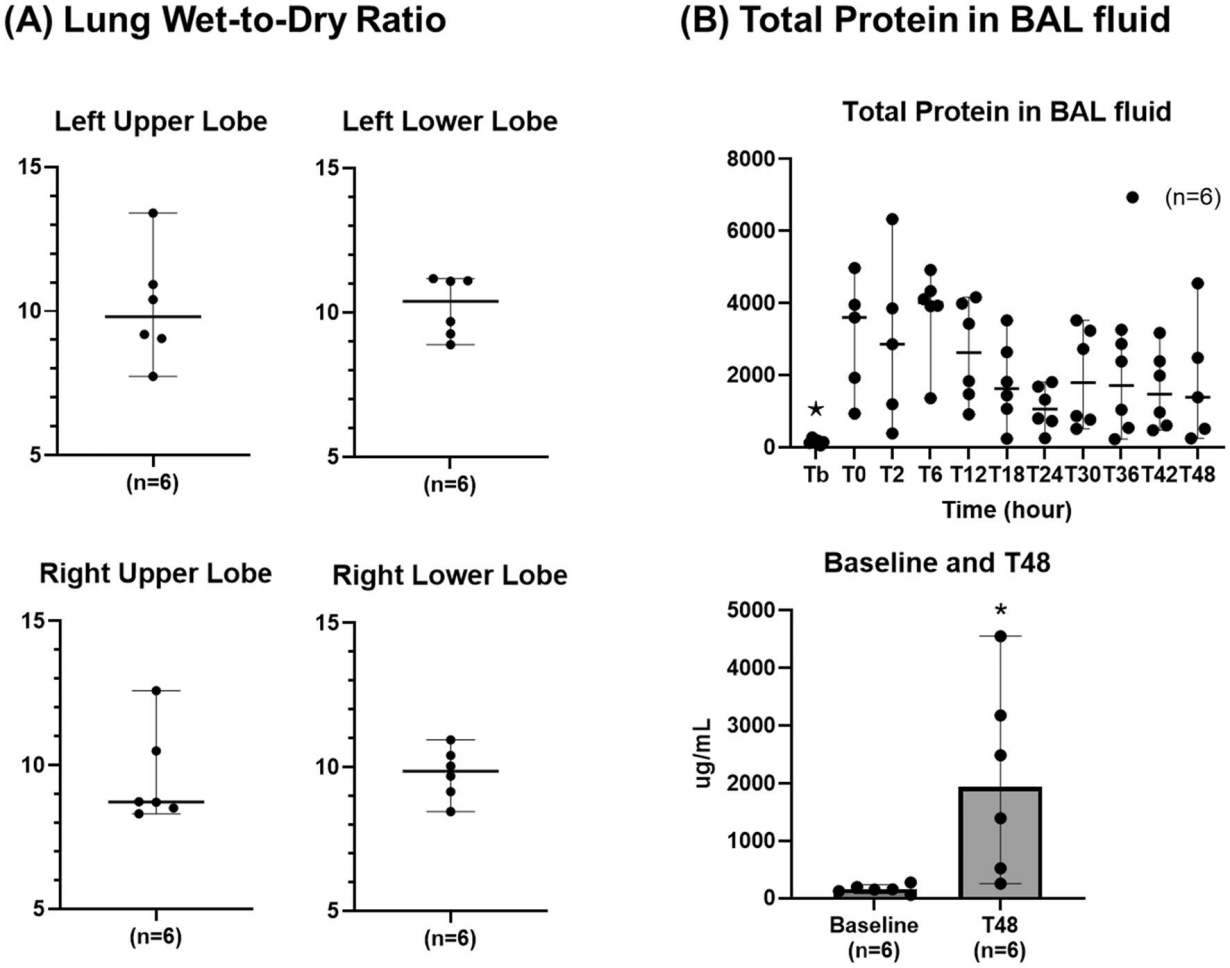
Alteration of the alveolar–capillary barrier. **A** Lung wet-to-dry ratio. **B** Total protein in BAL fluid. ⋆: *p* value < 0.05 with the Friedman test. _*: *p*_ value < 0.05 with the Wilcoxon signed-rank test. BAL: bronchoalveolar lavage

As shown in Fig. 8, the lung wet-to-dry ratios in the left upper (10.1 ± 0.8) and lower lobes (10.2 ± 0.4) and right upper (9.6 ± 0.7) and lower lobes (9.8 ± 0.4) increased. Those levels were significantly higher than the references in a previously published ECMO model without any injuries (Additional file 1: Fig. S8). The total protein level in the bronchoalveolar lavage (BAL) significantly increased after the injuries at T0 (3211 ± 607 ug/mL), reaching a peak level at T6 (3762 ± 504 ug/mL), decreasing over the following 18 h until T24 (1790 ± 475 ug/mL), and then persisting at a high level between T30 and T48. The total protein level in the BAL at T48 was significantly higher than the level observed at baseline before the development of the injuries (2062.7 ± 676.4 vs 157.6 ± 29.6 ug/mL, *p* = 0.02).

#### BAL assays

**Fig. 9:**
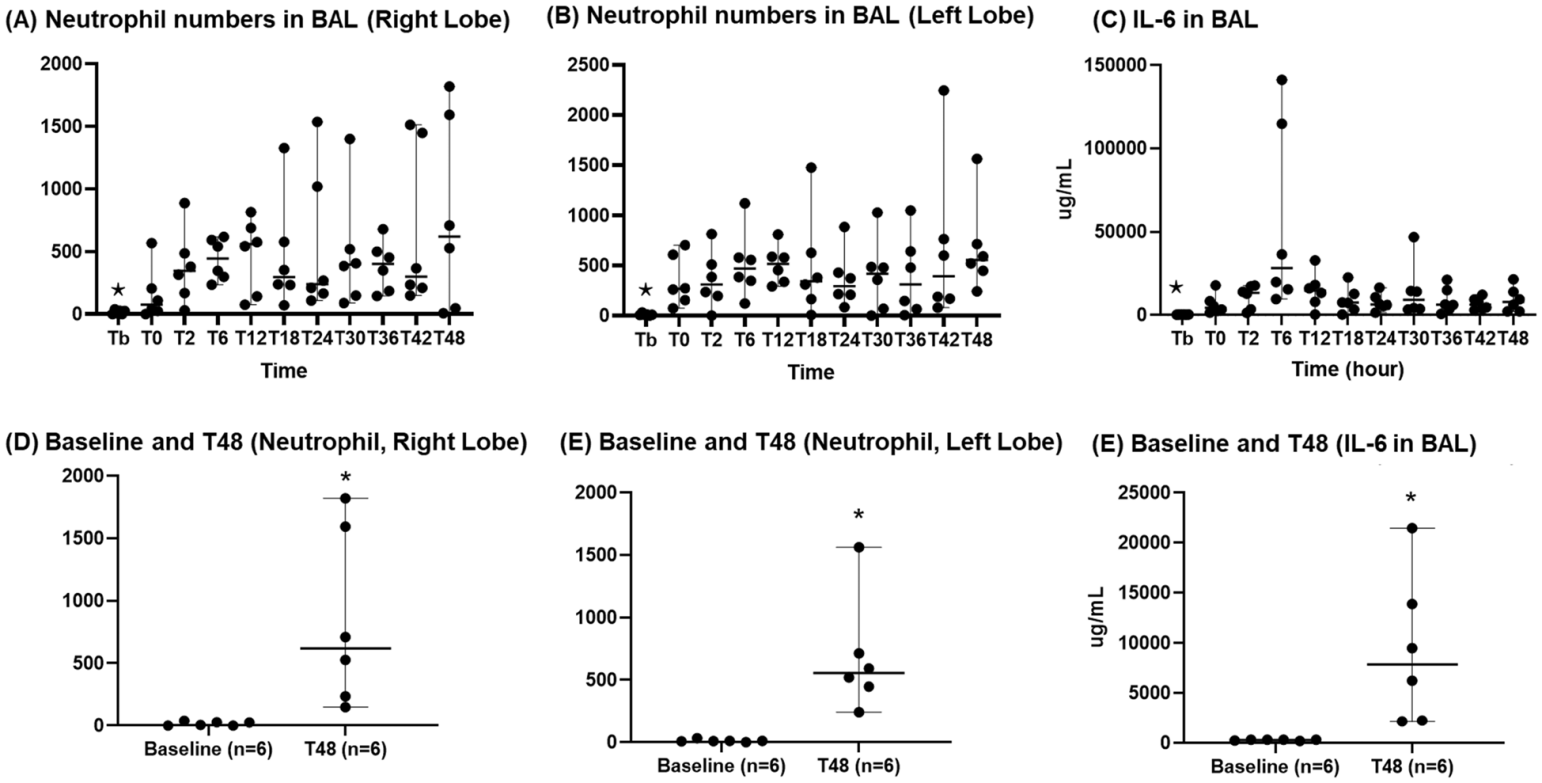
Presence of an inflammatory response. **A** Neutrophil numbers in BAL (right lobe), **B** neutrophil numbers in BAL (left lobe), **C** IL6 in BAL, **D** baseline and T48 (neutrophil, right lobe), **E** baseline and T48 (neutrophil, left lobe), **F** baseline and T48 (IL-6 in BAL). ⋆: *p* value < 0.05 with the Friedman test. *: *p* value < 0.05 with the Wilcoxon signed-rank test. BAL: bronchoalveolar lavage

As shown in Fig. 9, BAL neutrophils significantly increased in both the right and left lobes. In comparison with baseline values, quantitative neutrophils of the right lung BAL were 16 ± 7 vs. 839 ± 288, *p* = 0.03, and of the left lung were 12 ± 4 vs 680 ± 188, *p* < 0.01, respectively. IL-6 increased after the injuries, peaked at T6 (56,205.2 ± 23,240.7 pg/ml), and then showed consistently increased levels between T12 and T48. The level of IL-6 at T48 was significantly higher than the baseline values (9245.2 ± 3051.4 pg/ml vs 280.9 ± 20.6 pg/ml, *p* = 0.01).

#### Gas exchanges and lung mechanics

**Fig. 10:**
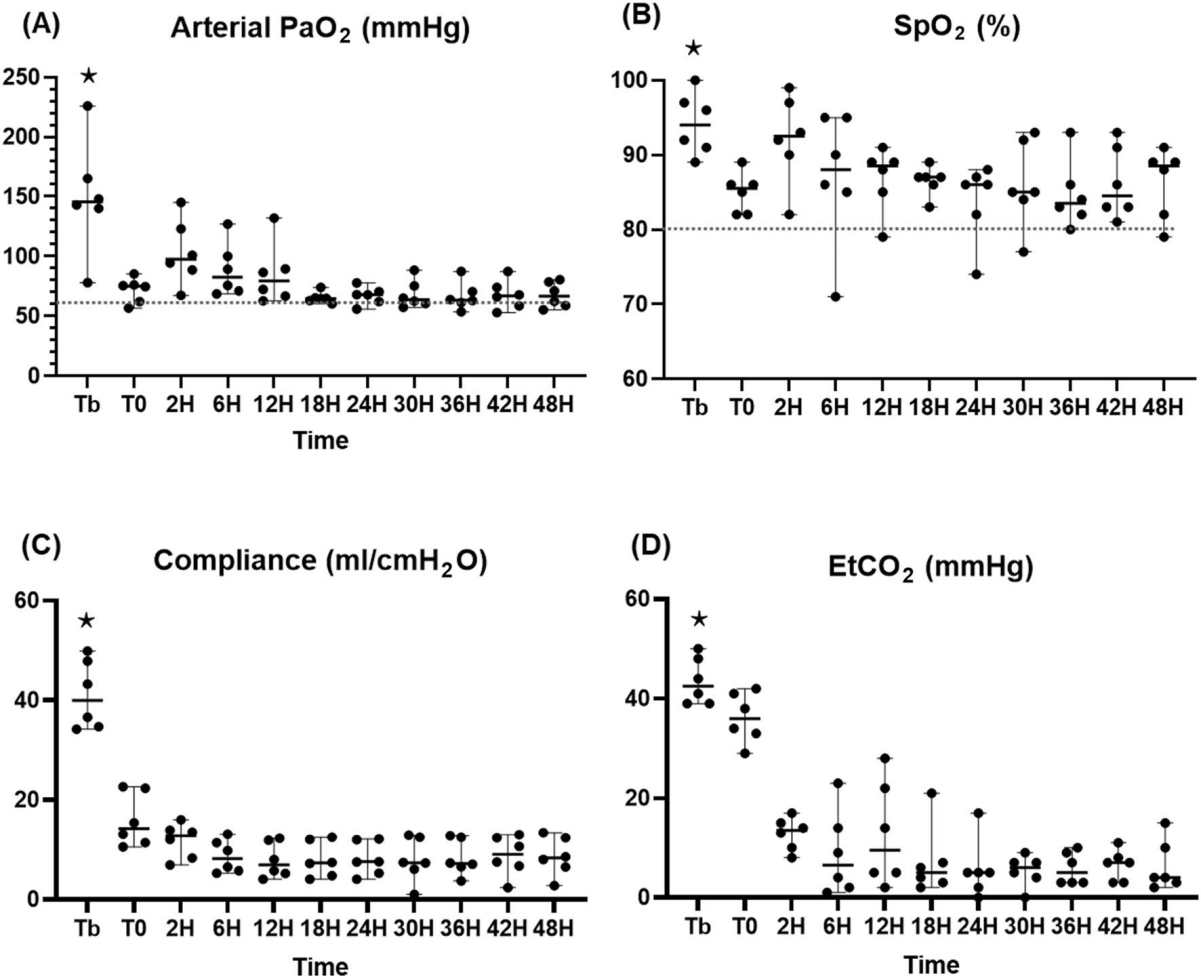
Evidence of physiological dysfunction. **A** Arterial PaO_2_ (mmHg), **B** SpO_2_ (%), **C** compliance (ml/cmH_2_O), **D** EtCO_2_ (mmHg). ⋆: *p* value < 0.05 with the Friedman test

We detailed gas exchanges and lung mechanics in Fig. 10. Significant decreases, from the baseline, in gas exchanges such as PaO_2_ and SpO_2_ and in lung mechanics such as compliance of the lungs, were observed until T6 and then maintained at a low level with a mean of 75.5 ± 4.5 cmH_2_O, 88.6 ± 0.8%, and 8.7 ± 0.4 ml/cmH_2_O, respectively. The EtCO_2_ level also deteriorated as the compliance decreased.

#### Cardiac function

**Fig. 11:**
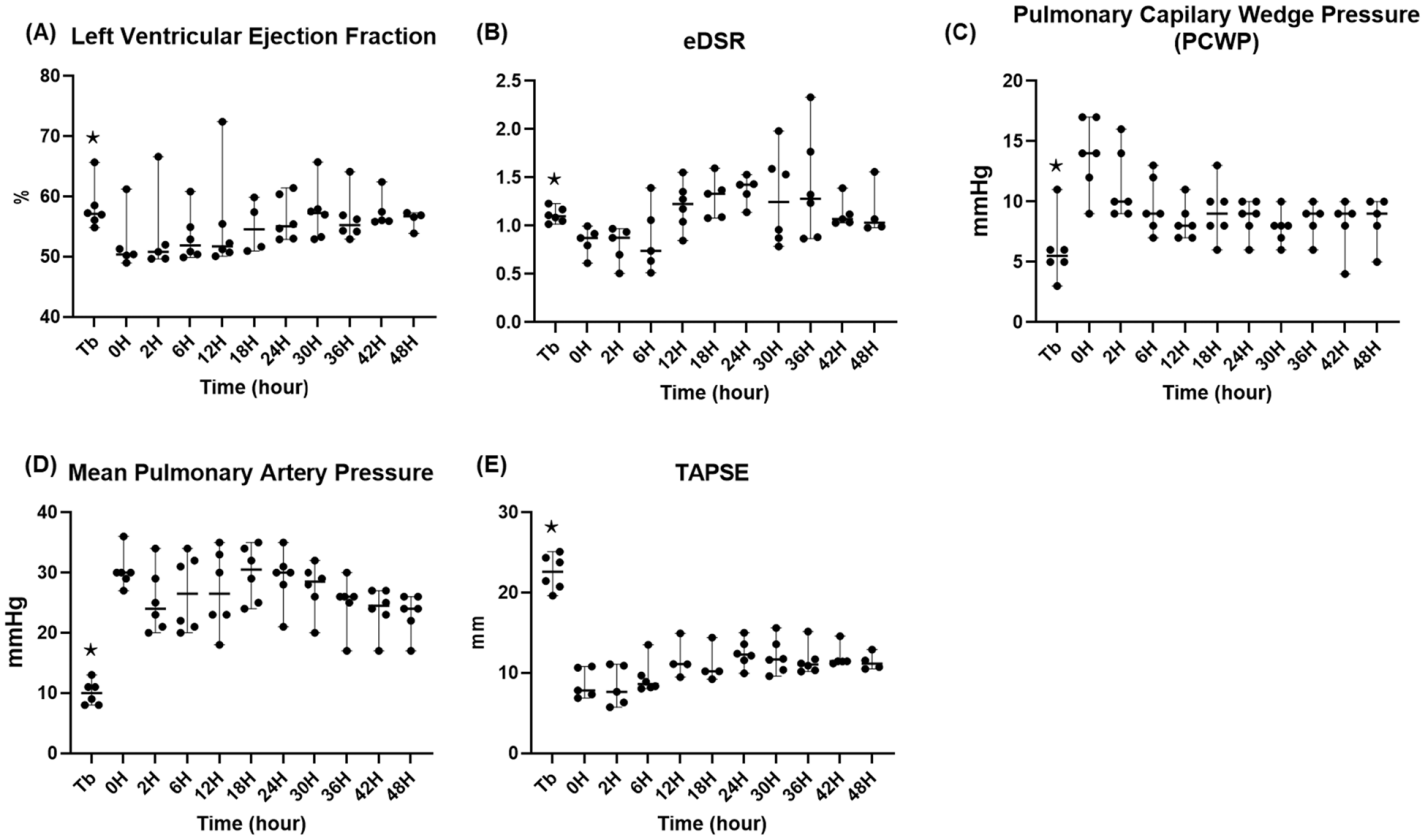
Cardiac function. ⋆: *p* value < 0.05 with the Friedman test. TAPSE; tricuspid annular plane systolic excursion

As shown in Fig. 11***,*** throughout the entire observation period, the left ventricular ejection fraction was 55.4 (± 0.5) %. The end-diastolic strain rate was in the normal range at Tb (1.1 ± 0.03), decreased for the first 6 h after the injury phases to below 1.0, then was restored and maintained in the normal range (1.0–1.5) until T48. The pulmonary capillary wedge pressure (PCWP) indicated that the left ventricular filling pressure increased at T0 (13.8 ± 1.3 mmHg) and then decreased despite fluid retention (8.4 ± 0.9 mmHg at T48). In response to the injury, the mean pulmonary artery pressure increased with average values of 30.3 ± 1.2 mmHg at T0, then gradually decreased (23.2 ± 1.4 mmHg at T48), while the tricuspid annular plane systolic excursion deteriorated to 8.7 ± 0.8 mmHg at T0, then gradually improved up to 11.4 ± 0.5 mmHg at T48.

## Discussion

This model-development study characterized an ovine model of refractory severe ARDS requiring VV-ECMO support, in which a clinically relevant ventilatory strategy was incorporated while developing the ARDS injury. Two important results need to be highlighted. First, the ventilatory strategy—featuring precise PEEP/FIO₂ titration and the avoidance of excessive airway pressures—led to an improvement in respiratory function, progressing from moderate to mild ARDS. This highlights the risks of overestimating the severity of injury during early disease development, potentially leading to erroneous conclusions in interventional studies. Second, the suitability of the developed model as an experimental ARDS model was confirmed across the four aspects proposed by the ATS Workshop Report for preclinical ARDS. Finally, no evidence of left ventricular failure was observed in the animals, confirming that lung deterioration was not confounded by concomitant cardiac failure.

Existing animal models of ARDS often fail to accurately mimic the complexity and variability of human ARDS, leading to challenges in translating preclinical findings into clinical practice[1, 9, 14, 15]. Indeed, many therapeutic agents that showed promising results during preclinical experiments failed to show any clinical benefits [1, 22, 23]. One of the major issues is the achievement of high-severity lung injury [16] and the lack of standardized mechanical ventilation management [15], which could significantly impact resulting outcomes. As demonstrated in this study, the ventilatory strategy with fine-tuning of PEEP settings, protective ventilation, and neuromuscular blockade, in line with clinical practice guidelines [17, 24], significantly improved the severity of lung injury (Fig. 3A). This was also highlighted in our previous paper, comparing the fixed mechanical ventilation strategy conventionally used in ARDS animal models with the adjustable lung-protective ventilation following the current guidelines and resulting in a significant difference in the achieved lung injury during the acute phase of ARDS [16]. This was confirmed by the need for further insults to achieve an indication for VV-ECMO. Our methods mirror clinical management, where ventilation strategies are continuously adjusted based on patient response and ARDS progression. Additional file 1: Fig. S8 also underscored the importance of continuous and dynamic adjustments in ventilator settings in preclinical studies. Importantly, a systematic review demonstrated that previous animal models of ARDS with ECMO support only employed static PEEP levels (i.e. 2–8 cmH_2_O of PEEP) [25–27]. Furthermore, these models frequently relied on non-clinically relevant ECMO indication criteria (i.e. 150–300 mmHg of PaO_2_/FIO_2_ ratio) and PEEP levels (5–10 cmH_2_O) [26–29], to ensure survival of the animal during model development. However, introducing ECMO in cases of insufficient severity of ARDS not only fails to improve outcomes [6], but may also cause adverse events, worsening their prognosis [30, 31], thereby losing the translatability and applicability of the findings into clinical practice. To the best of our knowledge, this is the first study to incorporate a clinically relevant ventilatory strategy in an ARDS model requiring VV-ECMO.

Human ARDS is caused by a variety of etiologies, each characterized by distinct pathophysiological mechanisms [32, 33 replicating the complexity of ARDS pathophysiology in preclinical experiments is challenging. In this context, the ATS Workshop Report provides essential guidance to prevent overlooking critical features of ARDS and ensure translatability [14]. For example, the diffuse alveolar damage (DAD) pattern, once regarded as a hallmark of human ARDS and widely replicated in preclinical models [34], is now recognized through human autopsy studies to be present in only 45–66% [35]. This finding has prompted a reassessment of the diagnostic value of DAD. Furthermore, the development of ARDS is a complex, multifaceted process characterized by several key phenomena, such as damage to the alveolar–capillary barrier, atelectasis, intra-alveolar accumulation of protein-rich fluid and inflammatory cells, and ventilation/perfusion mismatch [36]. Collectively, these factors lead to impaired gas exchange [37]. However, the clinical diagnosis of ARDS often focuses primarily on gas exchange impairment [38], neglecting the underlying pathophysiological mechanisms. In contrast, our study offers a comprehensive approach by incorporating both mechanistic and clinical parameters to validate the development of severe ARDS, a consistency that was maintained throughout the study.

Cardiac function should also be closely monitored during preclinical ARDS experiments, since left ventricular failure can be misdiagnosed as ARDS if not investigated appropriately [39]. Of note, cardiac function has rarely been investigated in preclinical ARDS experiments. In our study, we found that left ventricular function was within the normal range throughout the follow-up period (Additional file 1: Fig. S11), ensuring that our lung function results were not biased by a failing heart. Importantly, the impact of ARDS development, through OA and LPS, on right ventricular dysfunction should be emphasized [40]. Since left ventricular function was preserved, our model presents an additional application to study RV injury, management and recovery during ARDS.

A key finding is that all animals successfully survived the acute phase of refractory severe ARDS, an outcome that would not have been possible without extracorporeal support. In this double-hit model, the animals developed profound hypoxaemia (PaO_2_ < 100mmHg), progressive hypercapnic acidosis (PaCO_2_ > 50mmHg), and haemodynamic instability as shown in our findings. Without VV-ECMO support, as reported in previous studies by Wildi et al. [ref], this would have resulted in life-threatening acidosis, severe right ventricular failure due to pulmonary hypertension, systemic hypoperfusion, lactic acidosis, acute kidney injury which would have worsened metabolic acidosis, and ultimately multi-organ failure. The role of VV-ECMO in this model was therefore not merely supportive but essential for maintaining adequate oxygen delivery and carbon dioxide removal, thereby preventing the cascade of respiratory acidosis, renal dysfunction, and circulatory collapse. Additional file 1: Fig. S9 illustrates that ECMO parameters required continuous adjustment according to gas exchange and haemodynamic status over the 48-h period of observation, highlighting the dynamic interaction between extracorporeal support and evolving animal conditions. Compared with previous reports in which severe ARDS models without extracorporeal support showed high early mortality due to refractory hypoxemia and acidosis, our 100% survival rate underscores the physiological necessity of VV-ECMO in sustaining this severe injury model long enough to evaluate disease progression and therapeutic interventions. In addition to adherence to international ARDS management standards, the structured multidisciplinary ECMO team approach—advocated by the Extracorporeal Life Support Organization (ELSO)—was fundamental [41]. ECMO requires fine adjustment of several parameters to avoid complications, such as haemodynamic compromise, bleeding, or thrombosis associated with inappropriate blood flow, sweep gas flow, or anticoagulation management. Thus, the pre-experimental simulation training and post-experimental debriefing sessions under the supervision of an experienced ECMO director likely contributed to optimizing extracorporeal support and preventing secondary complications.

We recognize both the strengths and limitations of this study. First, this was an experimental study to develop a novel model of ARDS supported by VV-ECMO, without comparisons or controls with previous similar models. Due to the lack of consistency in injury strategy with previous ones, comparing the results with previous models is not appropriate to evaluate our findings. However, we believe this study provides essential insights that can inform changes to preclinical practices and settings, ultimately advancing this field and improving the translatability of preclinical findings into clinical applications. Second, this was a model development study carried out in only a few subjects. However, despite the limited sample size with varied responses to the ventilatory strategy and subsequent injuries (Figs. 2 and 3), the strength of our findings lies in consistency in terms of feasibility, safety, and clinical relevance. The sample size needed to assess the effects of an intervention should be calculated in future preclinical studies, especially for interventional studies. In addition, it would be valuable to test if the ventilatory strategy shows similar responses in different injury cohorts to validate our findings. Third, this study appraised only a limited number of variables. While we intentionally restricted these measurements to support the core concepts of the study, in future studies, a broader array of measurements needs to be performed based on the pathophysiological cascades that the intervention is targeting. Fourth, the double-hit injury model using OA and LPS has been previously validated by our group [42], and it is important to emphasize that its strength lies in producing stronger, systemic, and persistent injury. In contrast, other double-hit injury models, i.e. lung lavage and injurious ventilation are consistent with atelectasis-related biotrauma and subsequent volu/baro-trauma. Since viral and bacterial pneumonia are the most common clinical risk factors for ARDS, the inflammatory pathophysiological features of the OA + LPS model may provide superior generalizability and translatability. In addition, both OA and LPS can induce pulmonary hypertension, a feature commonly observed in patients with severe ARDS but difficult to reproduce in other experimental models of lung injury. Fifth, the 48-h follow-up period was limited to characterizing only the early phase of ARDS. Future studies should extend the observation period beyond 48 h to capture the later stages of ARDS progression. It would also be of interest to focus on other organs, such as the kidney or liver, using the same ARDS model under ECMO support and a similar long observational period, as these aspects have not been investigated under ECMO support.

## Conclusions

We developed a novel ovine model of severe ARDS requiring VV-ECMO support, incorporating a clinically relevant ventilatory strategy during lung injury. Our findings have proven that the model is feasible, safe, easily reproducible, and suitable for experimental ARDS injury. The approaches proposed in this study address some of the critical issues associated with current animal models and offer a robust platform for future research aimed at improving the treatment and management of ARDS.

## Supplementary Information

Additional file 1.

## Data Availability

The datasets used and/or analysed during the current study are available in Additional file 1: Table S4 or from the corresponding author upon reasonable request.

## Abbreviations

ABG: Arterial blood gas
ALI: Acute lung injury
ANOVA: Analysis of variance
ARDS: Acute respiratory distress syndrome
ATS: American Thoracic Society
BAL: Broncho-alveolar lavage
CT: Computed tomography
DAD: Diffuse alveolar damage
ECMO: Extracorporeal membrane oxygenation
ELSO: Extracorporeal life support organization
FIO_2_: Fraction of inspired oxygen
IL: Interleukin
LPS: Lipopolysaccharide
OA: Oleic acid
PCWP: Pulmonary capillary wedge pressure
PEEP: Positive end-expiratory pressure
RR: Respiratory rate
SARS-CoV-2: Severe acute respiratory syndrome coronavirus 2
SEM: Standard error of mean
VV-ECMO: Venovenous extracorporeal membrane oxygenation
