# Supplementary material for: Refining an acute respiratory distress syndrome animal model supported by extracorporeal membrane oxygenator: incorporating clinically relevant mechanical ventilation strategy upon development
Source: Intensive Care Med Exp. 2026 Apr 20;14:49. doi: 10.1186/s40635-026-00892-7 (PMC13096414; doi:10.1186/s40635-026-00892-7)
Supplement: Supplementary file 1 — Additional file 1. [file 40635_2026_892_MOESM1_ESM.docx]

**Refining an Acute Respiratory Distress Syndrome Animal Model Supported by Extracorporeal Membrane Oxygenator: Incorporating Clinically Relevant Mechanical Ventilation Strategy upon Development**

A running title: Essence to Optimise Experimental Acute Lung Injury

Keibun Liu^1,2,3^, Gabriele Fior^1,2^, Nchafatso Obonyo^1,2^, Hideaki Nonaka^1,2^, Angelo Milani^1,2^, Gabriella Abbate^1,2^, Sofia Portatadino^1,2^, Chiara Palmieri^4^, Shinichi Ijuin^1,2^, Kei Sato^1,2^, Silver Heinsar, Sun Kyun Ro^1,2^, Lucia Guandini^1,2^, Kota Hoshino^1,2^, Noriko Sato^1,2^, Samia Farah^1,2^, Carmen Aniola^1,2^, Margaret Passmore^1,2^, Rachana Pandura^1,2^, Mahe Bouquet^1,2^, Emily Wood^1,2^, Kieran Hyslop^1,2^, Molly-rose McInerney^1,2^, Cheng Zhang^1,5^, Caitlin McGrath^1,5^, Joshua Paech^6^, Gianluigi Li Bassi^1,2^, Jacky Suen^1,2,7,8^, and John Fraser^1,2,6,9,10^

1 Critical Care Research Group, The Prince Charles Hospital, Brisbane, Australia

2 Institute for Molecular Bioscience, The University of Queensland, Brisbane, Australia

3 Non-Profit Organization ICU Collaboration Network (ICON), Tokyo, Japan

4 The University of Queensland, School of Veterinary Science, Gatton, Australia.

5 Faculty of Medicine, The University of Queensland, Brisbane, Australia

6 St. Andrews War Memorial Hospital, Brisbane, Australia

7 School of Biomedical Sciences, Faculty of Medicine, University of Queensland, Brisbane, Australia

8 School of Pharmacy and Medical Sciences, Griffith University, Southport, Australia

9 Adult Intensive Care Services, The Prince Charles Hospital, Brisbane, Australia

10 Queensland University of Technology, Brisbane, Australia

**List of Online Supplemental Materials**

Supplemental Table 1: Animal Selection and Preparation

Supplemental Table 2: Coagulation management protocol during ECMO support

Supplemental Table 3: Safety assessments

Supplemental Table 4: Supplemental Table. Complete dataset of each variable.

Supplemental Figure 1: Mechanical ventilation management protocol

Supplemental Figure 2: Hemodynamic management protocol

Supplemental Figure 3: Protocol to develop refractory severe ARDS

Supplemental Figure 4: ECMO Indication Criteria

Supplemental Figure 5: ECMO management protocol

Supplemental Figure 6: Ultra-protective mechanical ventilation protocol

Supplemental Figure 7: The sampling time of the experiment

Supplemental Figure 8: Comparison of lung wet-to-dry ratio

**Supplemental Table 1. Animal Selection and Preparation**

| **Study information** | This was an observational preclinical study at the Medical Engineering Facility (MERF, the Queensland University of Technology, Brisbane) with ethics approvals from the University Animal Ethics Committee of the Queensland University of Technology (QUT) (2021000260) and the University of Queensland (2022/AE000077), following the ARRIVE guidelines. |
| --- | --- |
| **Animal Selection** | This study will use South African Mutton Merino (SAMM)/Leicester cross ewes aged 1-3 years. Animals will be sourced from the Warwick Saleyards, McEvoy Street/Bracker Road, Warwick, QLD and transferred to the MERF Pinjarra Hills farm. Animals will be inducted at Pinjarra Hills before transfer to QUT MERF. Sheep will be carefully monitored by veterinarians and have a period of acclimatisation (minimum 48 hours) at QUT MERF before inclusion in the study. |
| **Animal Preparation** | All animals were used after approval by the experienced veterinarian at MERF under a comprehensive and satisfactory pre-operative health assessment (e.g., no fever, no sign of infection, ect.) and full blood count (e.g., no anaemia, etc.). Before experimentation, all sheep were fasted overnight with free access to water. |
| **Inclusion criteria** | - Weight ≥ 40 kg but ≤ 70 kg  - Satisfactory pre-operative health assessment  - Fasted overnight |
| **Study team** | The study team conducting the experiments comprises an ECMO director, intensivists, emergency physicians, anesthesiologists, cardiovascular surgeons, cardiologists, pediatric physicians, and registered nurses. Before the study initiation, the ECMO director provided the team with education courses for ECMO basics and management and performed simulation training. |

MERF: Medical Engineering Facility, QLD: Queensland, QUT: Queensland University of Technology

**Supplemental Table 2. Coagulation management protocol during ECMO support**

| **Coagulation management** | |
| --- | --- |
| Protocol | Activated clotting time (ACT) of 180 – 220 seconds was targeted. At T0, ACT was measured to see if a bolus of 60 units/kg of heparin was necessary before the initiation of continuous heparin infusion at T1.  Continuous heparin infusion was started at 8 units/kg/hr of heparin (T1).  ACT was measured to monitor at T2, T4, T8, T16, T24, T32, T40, T48, and when necessary, and adjusted according to the titration table below. |
| **ACT (second)** | **Reaction** |
| < 130 | A bolus of 10 units/kg of heparin and increase infusion by 1.5 units/kg/hr |
| 130 - 150 | Increase infusion by 1 unit/kg/hr |
| 150 - 180 | Increase infusion by 0.5 units/kg/hr |
| 180 - 220 | No change |
| 220 – 250 | Decrease infusion by 1 unit/kg/hr |
| > 250 | Cease infusion for 1 hour and re-check ACT, restart when ACT < 220 seconds and decrease infusion by 1.5 units/kg/hr |
| **Electrolytes management** | |
| Potassium | Target: >3.5 and <5.5mmol/L  If <3.5mmol/L: apply potassium chloride (10 mmol/10 mL) continuous infusion  If >5.5mmol/L: Change the maintenance to Saline, Furosemide (20mg) injection, and Glucose-Insulin treatment: 5-10ml/h (10 units of regular insulin in 50 mL of 50 percent dextrose (25 g of glucose)) |
| Glucose | Target: >4 and <10mmol/L |
| Calcium | Target: > 1.00 mmol/L  If < 1.00 mmol/L with hemodynamic instability: bolus of 10ml Calcium gluconate |

ACT: activated clotting time

**Supplemental Table 3. Safety assessments**

| **Event** | **Definition** |
| --- | --- |
| **Bleeding complications** | |
| Cannulation site bleeding | Bleeding from a peripheral cannulation site such as the neck, groin, or axilla. Requiring PRBC transfusion (>20 mL/kg/24 h of PRBCs or > 3U PRBCs/24 h in adults) and/or surgical intervention (including intravascular hemostatic agent deployment). A reperfusion cannula is a type of peripheral cannulation site. |
| Surgical site bleeding | Bleeding from a surgical site other than a mediastinal or peripheral cannulation site requiring PRBC transfusion (>20 mL/kg/24 h of PRBCs or > 3U PRBCs/24 h in adults) and/or surgical intervention. |
| Tamponade | Tamponade during ECMO run requiring pericardial drain or mediastinal washout. |
| Pulmonary haemorrhage | Requiring PRBC transfusion (>20 mL/kg/24 h of PRBCs or > 3U PRBCs/24 h in adults). |
| Gastrointestinal bleeding | Upper or lower GI haemorrhage requiring PRBC transfusion (>20 mL/kg/24 h of PRBCs or > 3U PRBCs/24 h in adults) and/or endoscopic intervention and/or haemostatic agent deployment. |
| Hemorrhagic stroke | Intra/extra parenchymal central nervous system haemorrhage (may be intraparenchymal, subdural, or subarachnoid) or >= Grade 2 intraventricular haemorrhage seen on CT |
| **Thrombosis complications** | |
| Clots in the circuit | Clot formation or mechanical failure requiring a change of a circuit component (e.g. pigtails, connectors, bridge, arterial or venous tubing). |
| Oxygenator failure | Requiring change due to clot formation gas exchange failure or blood leak. |
| Pump failure | Failure of the pump requires hand cranking or pump exchange. |
| Hemolysis | Peak plasma-free haemoglobin ≥50 mg/dL occurring at least once during the ECMO run and sustained for at least 2 days or leading to a major component change (membrane lung, blood pump, entire circuit). |
| Ischemic stroke | Central nervous system infarction with CT, demonstrating localized ischemic change. |
| **ECMO performances** | |
| Circulation | Flow rate, rotation per minute (RPM), pressure P1 (pre-centrifuge pump), P2 (pre-oxygenator), and P3 (post-oxygenator), and) |
| Oxygenator performance | ECMO flow rate/RPM, resistance within oxygenator membrane: (P2-P3)/flow rate, sweep gas flow rate, gas exchange: Pre-PaCO_2_ – Post-PaCO_2_  Pre-PaCO_2_: partial pressure of carbon dioxide in the pre-oxygenator blood, Post-PaCO_2_: partial pressure of carbon dioxide in the post-oxygenator blood |
| Recirculation (%) | Recirculation of the ECMO circulation was calculated as follows: (SpreO_2_ – SvO_2_) / (SpostO_2_ – SvO_2_) × 100  SpreO_2_ = oxygen saturation of blood entering the oxygenator, SpostO2 = oxygen saturation of blood exiting the oxygenator, SvO2 = oxygen saturation of venous blood returning to the supra vena cava, which was drawn from the CV line in the left external jugular vein, just before being drained by the ECMO drainage cannula |

CT: computed tomography, ECMO: extracorporeal membrane oxygenation, PaCO2: partial pressure of carbon dioxide, PRBC: packet red blood cell,

**Supplemental Table 4. Complete dataset of each variable.**

| Variable | Sheep  Number | Tb | T0 | T1 | T2 | T6 | T12 | T18 | T24 | T30 | T36 | T42 | T48 |
| --- | --- | --- | --- | --- | --- | --- | --- | --- | --- | --- | --- | --- | --- |
| **Figure 5** | | | | | | | | | | | | | |
| ECMO Flow Rate (ml/kg/min) | 1 |  |  | 48.214286 | 46.428571 | 48.214286 | 48.214286 | 48.214286 | 53.571429 | 54.464286 | 55.357143 | 53.75 | 53.571429 |
|  | 2 |  |  | 59.4 | 60.2 | 61.6 | 62 | 62 | 54.8 | 57.6 | 56 | 55.6 | 55.8 |
|  | 3 |  |  | 58.333333 | 56.875 | 56.666667 | 58.333333 | 57.708333 | 58.125 | 58.333333 | 56.25 | 54.166667 | 53.125 |
|  | 4 |  |  | 42.8 | 42.8 | 50.6 | 56 | 54.6 | 56.6 | 56.4 | 57.2 | 56.4 | 55.8 |
|  | 5 |  |  | 49.090909 | 45.636364 | 34.909091 | 35.818182 | 48.363636 | 48.909091 | 49.818182 | 50 | 51.272727 | 52 |
|  | 6 |  |  | 47.719298 | 48.596491 | 51.578947 | 52.631579 | 52.45614 | 51.929825 | 52.105263 | 52.280702 | 52.280702 | 52.45614 |
| ECMO RPM | 1 |  |  | 2570 | 2570 | 2580 | 2360 | 2310 | 2520 | 2520 | 2520 | 2520 | 2530 |
|  | 2 |  |  | 2860 | 2860 | 2860 | 2860 | 2860 | 2700 | 2700 | 2700 | 2700 | 2700 |
|  | 3 |  |  | 2610 | 2600 | 2590 | 2670 | 2670 | 2670 | 2670 | 2670 | 2480 | 2420 |
|  | 4 |  |  | 2440 | 2490 | 2510 | 2500 | 2500 | 2500 | 2500 | 2500 | 2510 | 2510 |
|  | 5 |  |  | 2630 | 2540 | 2360 | 2410 | 2680 | 2640 | 2640 | 2640 | 2700 | 2700 |
|  | 6 |  |  | 2730 | 2740 | 2780 | 2780 | 2780 | 2770 | 2770 | 2780 | 2780 | 2770 |
| ECMO Pressure P1 (mmHg) | 1 |  |  | 10 | 3 | 0 | 10 | 14 | 9 | 8 | 11 | 8 | 3 |
|  | 2 |  |  | 0 | 0 | -1 | -1 | -2 | -13 | 0 | -1 | -11 | -11 |
|  | 3 |  |  | 4 | 5 | 1 | -4 | -5 | -7 | -5 | -9 | 6 | 9 |
|  | 4 |  |  | 4 | -3 | 12 | 16 | 9 | 16 | 15 | 17 | 17 | 17 |
|  | 5 |  |  | 0 | 2 | -15 | -15 | -2 | 8 | 9 | 9 | 6 | 7 |
|  | 6 |  |  | 7 | 6 | 7 | 10 | 11 | 12 | 10 | 10 | 10 | 10 |
| ECMO Pressure P2 (mmHg) | 1 |  |  | 151 | 150 | 155 | 152 | 150 | 169 | 171 | 173 | 170 | 165 |
|  | 2 |  |  | 215 | 216 | 214 | 213 | 212 | 180 | 191 | 190 | 180 | 180 |
|  | 3 |  |  | 183 | 184 | 178 | 183 | 182 | 181 | 182 | 176 | 166 | 161 |
|  | 4 |  |  | 158 | 160 | 176 | 179 | 171 | 178 | 178 | 179 | 180 | 180 |
|  | 5 |  |  | 180 | 170 | 131 | 137 | 188 | 188 | 190 | 189 | 196 | 196 |
|  | 6 |  |  | 205 | 204 | 212 | 213 | 214 | 215 | 211 | 212 | 213 | 213 |
| ECMO Pressure P3 (mmHg) | 1 |  |  | 120 | 116 | 123 | 120 | 119 | 137 | 136 | 141 | 139 | 135 |
|  | 2 |  |  | 178 | 178 | 176 | 176 | 176 | 148 | 158 | 158 | 149 | 148 |
|  | 3 |  |  | 155 | 155 | 153 | 155 | 154 | 153 | 155 | 151 | 143 | 138 |
|  | 4 |  |  | 111 | 108 | 117 | 125 | 120 | 126 | 126 | 126 | 126 | 126 |
|  | 5 |  |  | 154 | 142 | 104 | 109 | 150 | 152 | 155 | 156 | 161 | 162 |
|  | 6 |  |  | 169 | 168 | 177 | 180 | 183 | 181 | 182 | 181 | 181 | 180 |
| ECMO Flow Rate / RPM | 1 |  |  | 1.050584 | 1.011673 | 1.046512 | 1.144068 | 1.168831 | 1.190476 | 1.210318 | 1.230159 | 1.194444 | 1.185771 |
|  | 2 |  |  | 1.038462 | 1.052448 | 1.076923 | 1.083916 | 1.083916 | 1.014815 | 1.066667 | 1.037037 | 1.02963 | 1.033333 |
|  | 3 |  |  | 1.072797 | 1.05 | 1.050193 | 1.048689 | 1.037453 | 1.044944 | 1.048689 | 1.011236 | 1.048387 | 1.053719 |
|  | 4 |  |  | 0.877049 | 0.859438 | 1.007968 | 1.12 | 1.092 | 1.132 | 1.128 | 1.144 | 1.123506 | 1.111554 |
|  | 5 |  |  | 1.026616 | 0.988189 | 0.813559 | 0.817427 | 0.992537 | 1.018939 | 1.037879 | 1.041667 | 1.044444 | 1.059259 |
|  | 6 |  |  | 0.996337 | 1.010949 | 1.057554 | 1.079137 | 1.07554 | 1.068592 | 1.072202 | 1.071942 | 1.071942 | 1.079422 |
| Resistance within Membrane = Delta P/ BFR (mmHg) | 1 |  |  | 11.481481 | 13.076923 | 11.851852 | 11.851852 | 11.481481 | 10.666667 | 11.47541 | 10.322581 | 10.299003 | 10 |
|  | 2 |  |  | 12.457912 | 12.624585 | 12.337662 | 11.935484 | 11.612903 | 11.678832 | 11.458333 | 11.428571 | 11.151079 | 11.469534 |
|  | 3 |  |  | 10 | 10.622711 | 9.1911765 | 10 | 10.108303 | 10.035842 | 9.6428571 | 9.2592593 | 8.8461538 | 9.0196078 |
|  | 4 |  |  | 21.962617 | 24.299065 | 23.320158 | 19.285714 | 18.681319 | 18.374558 | 18.439716 | 18.531469 | 19.148936 | 19.354839 |
|  | 5 |  |  | 9.6296296 | 11.155378 | 14.0625 | 14.213198 | 14.285714 | 13.3829 | 12.773723 | 12 | 12.411348 | 11.888112 |
|  | 6 |  |  | 13.235294 | 12.99639 | 11.904762 | 11 | 10.367893 | 11.486486 | 9.7643098 | 10.402685 | 10.738255 | 11.036789 |
| ECMO Sweep Gas Flow Rate (L/min) | 1 |  |  | 3 | 3 | 2 | 2 | 5 | 4 | 4 | 3.5 | 4 | 4 |
|  | 2 |  |  | 3 | 2 | 2 | 3 | 3.5 | 3.5 | 3.5 | 3.5 | 3.5 | 4 |
|  | 3 |  |  | 3 | 3 | 3 | 3 | 3.5 | 3.5 | 3.5 | 3.5 | 3.5 | 3.5 |
|  | 4 |  |  | 3 | 3 | 4 | 4 | 4.5 | 5 | 5 | 5 | 5 | 5 |
|  | 5 |  |  | 3 | 2.5 | 2.5 | 4 | 10 | 10 | 8 | 8 | 6 | 6 |
|  | 6 |  |  | 2.5 | 2 | 3 | 2.5 | 3 | 3.5 | 4 | 3 | 3.5 | 3.5 |
| PaCO2pre-PaCO2post (mmHg) | 1 |  |  |  | 9.4 | 5.5 | 7.5 | 7.7 | 7.7 | 7.2 | 6.7 | 6.2 | 8 |
|  | 2 |  |  |  | 7.1 | 13.6 | 9.8 | 6.3 | 8.1 | 8.8 | 11.8 | 11 | 10.2 |
|  | 3 |  |  |  | 7 | 7.2 | 6.5 | 3.8 | 6.2 | 7.8 | 10 | 9.9 | 10.8 |
|  | 4 |  |  |  | 9.7 | 6.6 | 8.1 | 6.9 | 6.3 | 6.6 | 2.3 | 5.1 | 4.2 |
|  | 5 |  |  |  | 10.1 | 14.4 | 11.7 | 10.6 | 10 | 8.6 | 9.6 | 9.8 | 9.8 |
|  | 6 |  |  |  | 10.5 | 9.2 | 10.6 | 9.3 | 7.7 | 9.9 | 8.9 | 10 | 9.6 |
| Recirculation (%) | 1 |  |  |  | 15.333333 | 58.008658 | 37.192982 | 23.809524 | 4.5 |  |  |  |  |
|  | 2 |  |  |  | 78.883861 | 42.896936 | 38.802083 | 46.728972 | 35.091278 | 23.04038 | 30.5 | 13.296399 | 24.879227 |
|  | 3 |  |  |  | 71.064815 | 35.454545 | 30.483271 | 58.658009 | 66.900175 | 42.352941 | 32.126697 | 32.929293 | 34.606742 |
|  | 4 |  |  |  | 51.476793 | 40.697674 | 37.037037 | 51.002865 | 37.5 | 51.315789 | 54.754098 | 51.724138 | 44.88189 |
|  | 5 |  |  |  | 53.648069 | 18.670077 | 30.649351 | 16.587678 | 35.046729 | 22.636816 | 22.340426 | 25.835866 | 33.429395 |
|  | 6 |  |  |  | 77.583697 | 71.345029 | 66.470588 | 61.33829 | 45.480226 | 35.694823 | 28.476821 | 19.475655 | 24.06639 |
| Figure 6 | | | | | | | | | | | | | |
| PEEP (cmH_2_O) | 1 | 5 | 10 |  | 10 | 10 | 10 | 10 | 10 | 10 | 10 | 10 | 10 |
|  | 2 | 5 | 10 |  | 10 | 10 | 10 | 10 | 10 | 10 | 10 | 10 | 10 |
|  | 3 | 5 | 10 |  | 10 | 10 | 10 | 10 | 10 | 12 | 12 | 12 | 12 |
|  | 4 | 5 | 15 |  | 10 | 10 | 10 | 10 | 10 | 10 | 10 | 10 | 10 |
|  | 5 | 5 | 13 |  | 10 | 10 | 10 | 10 | 10 | 10 | 10 | 10 | 10 |
|  | 6 | 5 | 12 |  | 10 | 10 | 10 | 10 | 10 | 10 | 10 | 10 | 10 |
| Respiratory Rate (times per min) | 1 | 24 | 28 |  | 10 | 10 | 10 | 10 | 10 | 10 | 10 | 10 | 10 |
|  | 2 | 20 | 32 |  | 10 | 10 | 10 | 10 | 10 | 10 | 10 | 10 | 10 |
|  | 3 | 17 | 35 |  | 10 | 10 | 10 | 10 | 10 | 12 | 12 | 12 | 12 |
|  | 4 | 15 | 35 |  | 10 | 10 | 10 | 10 | 10 | 10 | 10 | 10 | 10 |
|  | 5 | 18 | 35 |  | 10 | 10 | 10 | 10 | 10 | 10 | 10 | 10 | 10 |
|  | 6 | 18 | 35 |  | 10 | 10 | 10 | 10 | 10 | 10 | 10 | 10 | 10 |
| Tidal Volume (ml/kg) | 1 | 5.910714 | 5.160714 |  | 2.75 | 2.839286 | 2.892857 | 2.839286 | 2.821429 | 2.803571 | 2.875 | 2.857143 | 2.785714 |
|  | 2 | 7.46 | 7.5 |  | 4.06 | 1.78 | 1.78 | 1.96 | 1.84 | 1.94 | 2 | 1.88 | 1.88 |
|  | 3 | 7.5 | 5.0416667 |  | 3.3541667 | 1.6458333 | 1.7083333 | 1.7083333 | 1.75 | 0.0895833 | 0.4583333 | 0.625 | 0.6666667 |
|  | 4 | 7.46 | 4.3 |  | 2.42 | 1.62 | 1.46 | 1.34 | 1.54 | 1.8 | 1.82 | 1.6 | 1.64 |
|  | 5 | 7.345455 | 5.036364 |  | 2.4 | 2.472727 | 2.2 | 1.545455 | 1.618182 | 1.654545 | 1.618182 | 1.563636 | 1.6 |
|  | 6 | 7.070175 | 5.368421 |  | 3.54386 | 2.719298 | 2.701754 | 1.964912 | 2.017544 | 2.105263 | 2.052632 | 2.105263 | 2.631579 |
| Plateau pressure (cmH2O) | 1 | 11.4 | 25.3 |  | 21.1 | 20.5 | 21 | 21.1 | 22.1 | 21 | 21.3 | 21 | 20.9 |
|  | 2 | 14.6 | 26.4 |  | 19.4 | 22.4 | 22.9 | 20 | 19.6 | 19.4 | 19.9 | 20.6 | 19.3 |
|  | 3 | 14.2 | 25.4 |  | 26.6 | 22.8 | 28.9 | 29.2 | 29.4 | 23.9 | 23.6 | 24 | 23.8 |
|  | 4 | 14 | 30.4 |  | 24.3 | 23.9 | 21.8 | 21.9 | 22 | 21.8 | 22.2 | 20.9 | 20.5 |
|  | 5 | 15.5 | 26.5 |  | 19.8 | 20.7 | 22 | 20.5 | 19.6 | 20.2 | 20.9 | 20.1 | 19.1 |
|  | 6 | 12.2 | 26 |  | 22.2 | 20.9 | 21.6 | 17.6 | 17.9 | 17.9 | 17.9 | 18.6 | 20.1 |
| Driving pressure (cmH2O) | 1 | 6.4 | 15.3 |  | 11.1 | 10.5 | 11 | 11.1 | 12.1 | 11 | 11.3 | 11 | 10.9 |
|  | 2 | 9.6 | 16.4 |  | 9.4 | 12.4 | 12.9 | 10 | 9.6 | 9.4 | 9.9 | 10.6 | 9.3 |
|  | 3 | 9.2 | 15.4 |  | 16.6 | 12.8 | 18.9 | 19.2 | 19.4 | 11.9 | 11.6 | 12 | 11.8 |
|  | 4 | 9 | 15.4 |  | 14.3 | 13.9 | 11.8 | 11.9 | 12 | 11.8 | 12.2 | 10.9 | 10.5 |
|  | 5 | 10.5 | 13.5 |  | 9.8 | 10.7 | 12 | 10.5 | 9.6 | 10.2 | 10.9 | 10.1 | 9.1 |
|  | 6 | 7.2 | 14 |  | 12.2 | 10.9 | 11.6 | 7.6 | 7.9 | 7.9 | 7.9 | 8.6 | 10.1 |
| Figure 8 | | | | | | | | | | | | | |
| Total Protein in BAL (ug/mL) | 1 | 152 | 935 |  | 2864 | 3918 | 3428 | 2645 | 1809 | 515 | 2384 | 608 | 519 |
|  | 2 | 125 | 1932 |  | 6332 | 4109 | 4165 | 3524 | 1325 | 2729 | 2869 | 967 | 2484 |
|  | 3 | 146 | 3959 |  | 1197 | 4335 | 919 | 1068 | 732 | 768 | 232 | 2389 | 1392 |
|  | 4 | 274 | 3605 |  | 393 | 1361 | 1835 | 243 | 804 | 867 | 543 | 475 | 253 |
|  | 5 | 56 | 4976 |  |  | 3929 | 1476 | 1441 | 257 | 3237 | 1045 | 3177 |  |
|  | 6 | 192 |  |  | 3859 | 4921 | 3993 | 1816 | 1682 | 3524 | 3266 | 1991 | 4552 |
| Figure 9 | | | | | | | | | | | | | |
| Neutrophil numbers in BAL (Right Lobe) | 1 | 26 | 0 |  | 887 | 593 | 816 | 239 | 166 | 520 | 680 | 149 | 8 |
|  | 2 | 0 | 206 |  | 380 | 347 | 690 | 354 | 109 | 1400 | 500 | 367 | 710 |
|  | 3 | 0 | 109 |  | 169 | 236 | 143 | 234 | 269 | 149 | 185 | 235 | 47 |
|  | 4 | 5 | 44 |  | 30 | 299 | 76 | 71 | 211 | 89 | 145 | 210 | 528 |
|  | 5 | 39 | 569 |  | 486 | 542 | 576 | 579 | 1536 | 407 | 454 | 1448 | 1820 |
|  | 6 | 27 | 25 |  | 315 | 618 | 544 | 1327 | 1019 | 386 | 351 | 1514 | 1594 |
| BAL left Neutrophil (absolute number) | 1 | 11 | 273 |  | 196 | 580 | 455 | 627 | 374 | 70 | 642 | 189 | 448 |
|  | 2 | 0 | 262 |  | 814 | 557 | 587 | 379 | 216 | 1029 | 480 | 170 | 592 |
|  | 3 | 9 | 73 |  | 386 | 386 | 292 | 165 | 84 | 359 | 5 | 601 | 520 |
|  | 4 | 8 | 608 |  | 2 | 348 | 338 | 8 | 210 | 0 | 66 | 80 | 242 |
|  | 5 | 10 | 154 |  | 510 | 122 | 810 | 311 | 429 | 480 | 147 | 764 | 714 |
|  | 6 | 32 | 705 |  | 236 | 1119 | 579 | 1475 | 886 | 486 | 1050 | 2245 | 1562 |
| Interleukin-6 in BAL (pg/mL) | 1 | 313 | 1478 |  | 13949 | 141131 | 313 | 313 | 16426 | 3368 | 21141 | 2976 | 2146 |
|  | 2 | 313 | 2415 |  | 12756 | 36493 | 32825 | 22613 | 6617 | 46873 | 14995 | 4523 | 9486 |
|  | 3 | 313 | 8283 |  | 1353 | 114832 | 13360 | 7329 | 6117 | 3632 | 728 | 7927 | 6243 |
|  | 4 | 312.5 | 3277.78 |  | 3394.83 | 9524.95 | 16041.4 | 3292.5 | 5280.86 | 4230.51 | 3298.17 | 3622.78 | 2248.07 |
|  | 5 | 202.6871 | 5379.289 |  | 17143.24 | 15395.83 | 7900.39 | 7610.507 | 1422.089 | 14417.87 | 6028.007 | 12128.76 | 21469.63 |
|  | 6 | 231.167 | 17799.6 |  | 17675.2 | 19854.3 | 18027.2 | 12709.6 | 10668.9 | 14014.7 | 6254.79 | 9309.99 | 13878.3 |
| Figure 10 | | | | | | | | | | | | | |
| Arterial PaO2 (mmHg) | 1 | 140 | 75.5 |  | 101 | 100 | 89.5 | 64.6 | 70.6 | 75.4 | 87.4 | 87.3 | 78.6 |
|  | 2 | 78.1 | 62.1 |  | 88.6 | 71.1 | 62.8 | 63.1 | 55.9 | 60.6 | 53.7 | 53.1 | 55.4 |
|  | 3 | 165 | 76.1 |  | 123 | 89.2 | 72.4 | 65.3 | 67.9 | 62.5 | 61.3 | 58.6 | 58.8 |
|  | 4 | 148 | 56.7 |  | 67.4 | 68.6 | 66.8 | 64.9 | 68.1 | 88.4 | 70.5 | 67.9 | 62.4 |
|  | 5 | 143 | 74.7 |  | 94.4 | 75.8 | 86.6 | 60.3 | 62.1 | 57.3 | 63.1 | 74 | 71.1 |
|  | 6 | 226 | 85.4 |  | 145 | 127 | 132 | 74 | 77.8 | 65.1 | 63.8 | 66.2 | 80.6 |
| SpO2 (%) | 1 | 92 | 82 |  | 93 | 95 | 88 | 87 | 87 | 92 | 93 | 93 | 88 |
|  | 2 | 89 | 85 |  | 92 | 86 | 85 | 89 | 86 | 85 | 82 | 83 | 79 |
|  | 3 | 96 | 86 |  | 99 | 95 | 91 | 87 | 88 | 85 | 83 | 81 | 82 |
|  | 4 | 100 | 86 |  | 97 | 90 | 89 | 86 | 86 | 93 | 86 | 86 | 89 |
|  | 5 | 97 | 89 |  | 90 | 85 | 89 | 83 | 82 | 84 | 80 | 91 | 89 |
|  | 6 | 91 | 82 |  | 82 | 71 | 79 | 87 | 74 | 77 | 84 | 83 | 91 |
| Compliance (ml/cmH2O) | 1 | 43.3 | 13.1 |  | 13.9 | 13.1 | 12.3 | 12.5 | 12 | 12.5 | 12.5 | 13 | 12.4 |
|  | 2 | 34.7 | 22.7 |  | 16 | 6.5 | 5.8 | 7.2 | 7.6 | 7.4 | 7.1 | 10.7 | 8.6 |
|  | 3 | 49.9 | 10.6 |  | 8.3 | 5.8 | 4.1 | 4.1 | 4.1 | 1 | 3.7 | 2.4 | 2.8 |
|  | 4 | 36.6 | 11.4 |  | 6.9 | 5.3 | 5.2 | 4.8 | 5.3 | 6.1 | 6.6 | 6.7 | 6.5 |
|  | 5 | 34.2 | 15.4 |  | 12.1 | 9.8 | 8 | 7.4 | 7.5 | 7.3 | 7.3 | 7.5 | 8 |
|  | 6 | 47.9 | 22.4 |  | 13.5 | 11.4 | 11.9 | 12.1 | 12.2 | 12.9 | 12.8 | 12.4 | 13.4 |
| EtCO2 (mmHg) | 1 | 41 | 42 |  | 8 | 9 | 14 | 7 | 0 | 7 | 7 | 7 | 4 |
|  | 2 | 39 | 29 |  | 17 | 2 | 2 | 2 | 2 | 4 | 3 | 3 | 4 |
|  | 3 | 44 | 38 |  | 15 | 4 | 5 | 6 | 5 | 0 | 3 | 3 | 3 |
|  | 4 | 39 | 34 |  | 10 | 1 | 5 | 3 | 5 | 7 | 10 | 7 | 2 |
|  | 5 | 50 | 33 |  | 13 | 23 | 28 | 4 | 5 | 5 | 3 | 11 | 10 |
|  | 6 | 48 | 41 |  | 14 | 14 | 22 | 21 | 17 | 9 | 9 | 8 | 15 |
| Figure 11 | | | | | | | | | | | | | |
| Left Ventricular Ejection Fraction (%) | 1 | 65.679995 | 61.228726 |  | 66.617996 | 60.811782 | 72.42529 |  | 55.407303 | 65.721403 | 64.127547 | 57.45 | 57.277953 |
|  | 2 | 56.99576 | 50.39875 |  | 49.686142 | 54.930337 | 50.098576 | 51.658346 | 53.005966 | 53.267579 | 54.313986 | 56.065593 | 56.585157 |
|  | 3 | 56.092637 | 48.975169 |  | 49.642947 | 49.886878 | 55.435614 |  | 60.392552 | 57.505825 | 52.910821 |  |  |
|  | 4 | 58.511895 |  |  |  | 50.840493 | 50.711453 | 59.838691 | 54.691579 | 57.923497 | 56.211444 | 62.427679 | 53.89574 |
|  | 5 | 54.864324 | 51.289871 |  | 50.816523 | 52.899197 | 52.242478 | 50.952381 | 52.871363 | 52.907348 | 54.188403 | 55.921053 | 56.86901 |
|  | 6 | 57.264767 | 50.22953 |  | 51.984053 | 50.369473 | 51.207898 | 57.428571 | 61.420213 | 56.975806 | 56.872038 | 55.807397 |  |
| End Diastolic Strain Rate | 1 | 1.0166667 | 0.9966667 |  | 0.8766667 | 0.74 | 1.2766667 | 1.09 | 1.53 | 1.9833333 | 1.7666667 | 1.07 | 1.56 |
|  | 2 | 1.05 | 0.8733333 |  | 0.97 | 1.3933333 | 1.3533333 | 1.5966667 |  | 0.7866667 | 0.8666667 | 1.03 | 0.9933333 |
|  | 3 | 1.0866667 | 0.9166667 |  | 0.5066667 |  | 1.1766667 |  | 1.14 | 0.8733333 | 2.3333333 |  |  |
|  | 4 | 1.11 |  |  |  | 0.6366667 | 1.0433333 | 1.08 | 1.33 | 1.59 | 1.3233333 | 1.12 | 0.98 |
|  | 5 | 1.23 | 0.7966667 |  | 0.9366667 | 1.06 | 1.5533333 | 1.37 | 1.4266667 | 0.96 | 0.8833333 | 1.0366667 | 1.07 |
|  | 6 | 1.17 | 0.6133333 |  | 0.7 | 0.5133333 | 0.8466667 | 1.3333333 | 1.4333333 | 1.5333333 | 1.2366667 | 1.39 |  |
| Pulmonary Capilary Wedge Pressure (PCWP) (mmHg) | 1 | 11 | 12 |  | 10 | 7 | 7 | 6 | 8 | 6 |  |  |  |
|  | 2 | 3 | 9 |  | 9 | 9 | 7 | 13 | 6 | 7 | 6 | 4 | 5 |
|  | 3 | 5 | 14 |  | 14 | 12 | 8 | 8 | 10 | 8 | 10 | 9 | 9 |
|  | 4 | 5 | 17 |  | 16 | 13 | 11 | 10 | 9 | 8 | 8 | 8 | 8 |
|  | 5 | 6 | 14 |  | 9 | 8 | 8 | 10 | 10 | 10 | 9 | 9 | 10 |
|  | 6 | 6 | 17 |  | 10 | 9 | 9 | 8 | 9 | 8 | 9 | 10 | 10 |
| Mean Pulmonary Artery Pressure (mmHg) | 1 | 8 | 30 |  | 20 | 20 | 23 | 24 | 21 | 20 | 17 | 17 | 17 |
|  | 2 | 13 | 27 |  | 29 | 34 | 33 | 29 | 28 | 28 | 26 | 23 | 22 |
|  | 3 | 9 | 29 |  | 25 | 32 | 30 | 32 | 31 | 26 | 26 | 27 | 26 |
|  | 4 | 11 | 36 |  | 34 | 31 | 35 | 35 | 35 | 32 | 30 | 27 | 26 |
|  | 5 | 11 | 30 |  | 23 | 22 | 18 | 25 | 30 | 29 | 25 | 24 | 24 |
|  | 6 | 8 | 30 |  | 21 | 21 | 23 | 34 | 30 | 30 | 26 | 25 | 24 |
| TAPSE (mm) | 1 | 24.35 | 10.8 |  | 10.91 | 8.21 |  |  | 15.02 | 10.39 | 11.2 | 11.45 | 10.74 |
|  | 2 | 25.11 | 7.33 |  | 5.74 | 8.36 | 11.12 | 10.21 | 11.59 | 11.63 | 10.18 | 11.46 | 10.52 |
|  | 3 | 20.76 | 10.65 |  | 6.35 | 9.69 |  |  | 9.97 | 9.61 | 11.71 |  |  |
|  | 4 | 21.46 |  |  |  | 8.07 | 11.1 | 9.23 | 12.16 | 11.75 | 10.91 | 11.18 | 12.92 |
|  | 5 | 19.65 | 7.84 |  | 7.66 | 8.91 | 9.49 | 10.21 | 12.4 | 15.6 | 10.34 | 11.46 | 11.56 |
|  | 6 | 23.78 | 6.88 |  | 11.1 | 13.5 | 14.95 | 14.4 | 13.58 | 13.59 | 15.16 | 14.59 |  |

**Supplemental Figure 1. Mechanical ventilation management protocol**


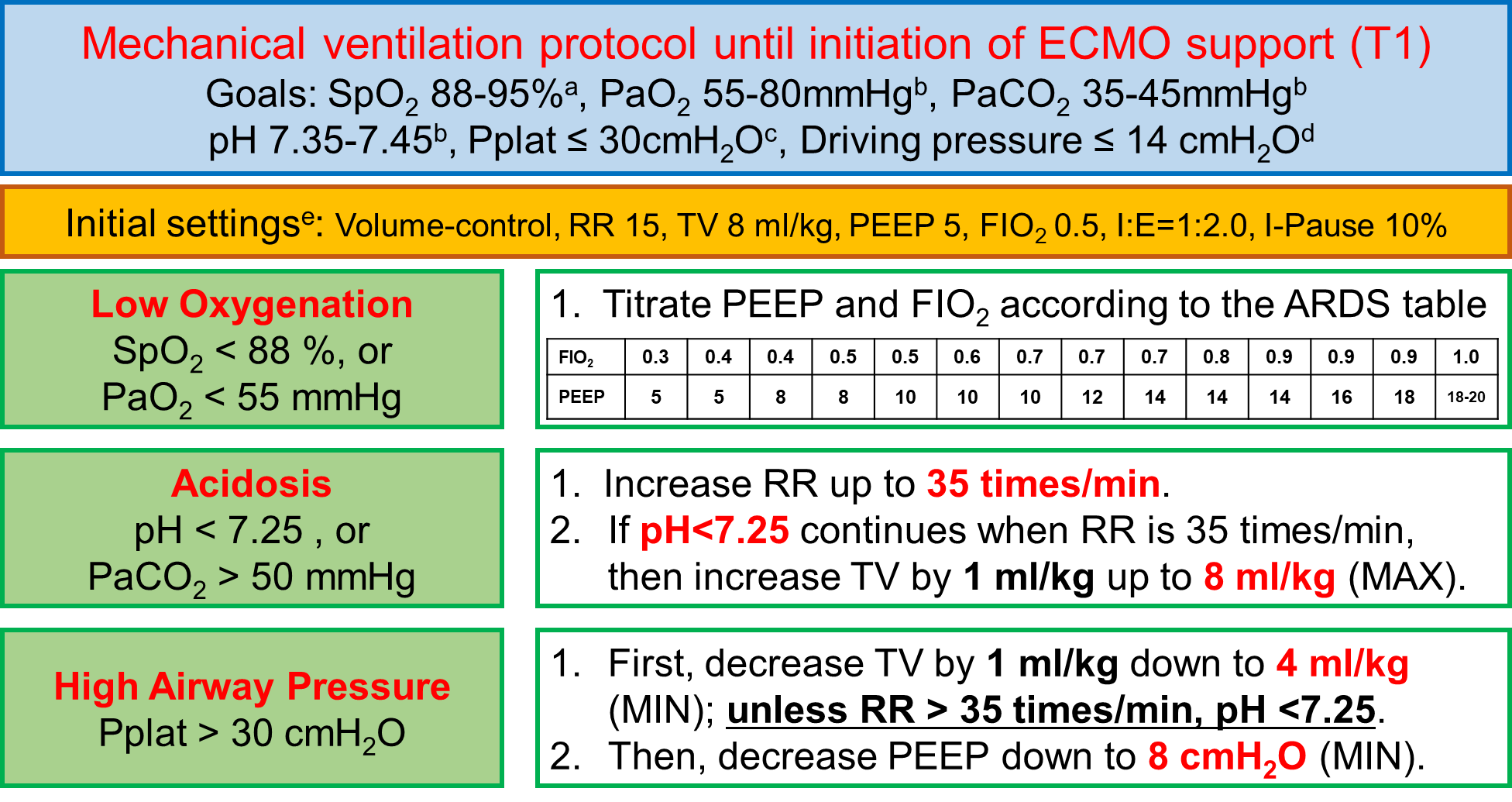


ARDS: acute respiratory distress syndrome, FIO_2_: fraction of inspired oxygen, I:E: inspiratory to expiratory ratios, I-Pause: inspiratory pause, MAX: maximum, MIN: minimum, PaCO_2_: partial pressure of carbon dioxide, PaO_2_: partial pressure of oxygen, PEEP: positive end-expiratory pressure, Pplat: plateau pressure, RR: respiratory rate, SpO_2_: percutaneous oxygen saturation, TV: tidal volume,

^a^ The percutaneous oxygen saturation was continuously monitored on the physiological monitor

^b^ These were measured by arterial blood gas.

^c^ The plateau pressure was measured on the mechanical ventilator.

^d^ The driving pressure was calculated as follows; (plateau pressure – PEEP).

^e^ The details of the initial settings were as follows; volume-controlled mode, tidal volumes of 8ml/kg, a PEEP of 5cmH20, a respiratory rate of 15 breaths/min, and FiO2 of 50%. The settings of the mechanical ventilation were adjusted based on the arterial blood gas results to maintain the following goals: PaO_2_ 55-80 mmHg, pH 7.35-7.45, and PaCO_2_ 35-45 mmHg in the arterial blood and SpO2 88-95%, and the end-tidal carbon dioxide (EtCO2) of 35-45mmHg on the physiological monitoring.

**Supplemental Figure 2. Hemodynamic management protocol**


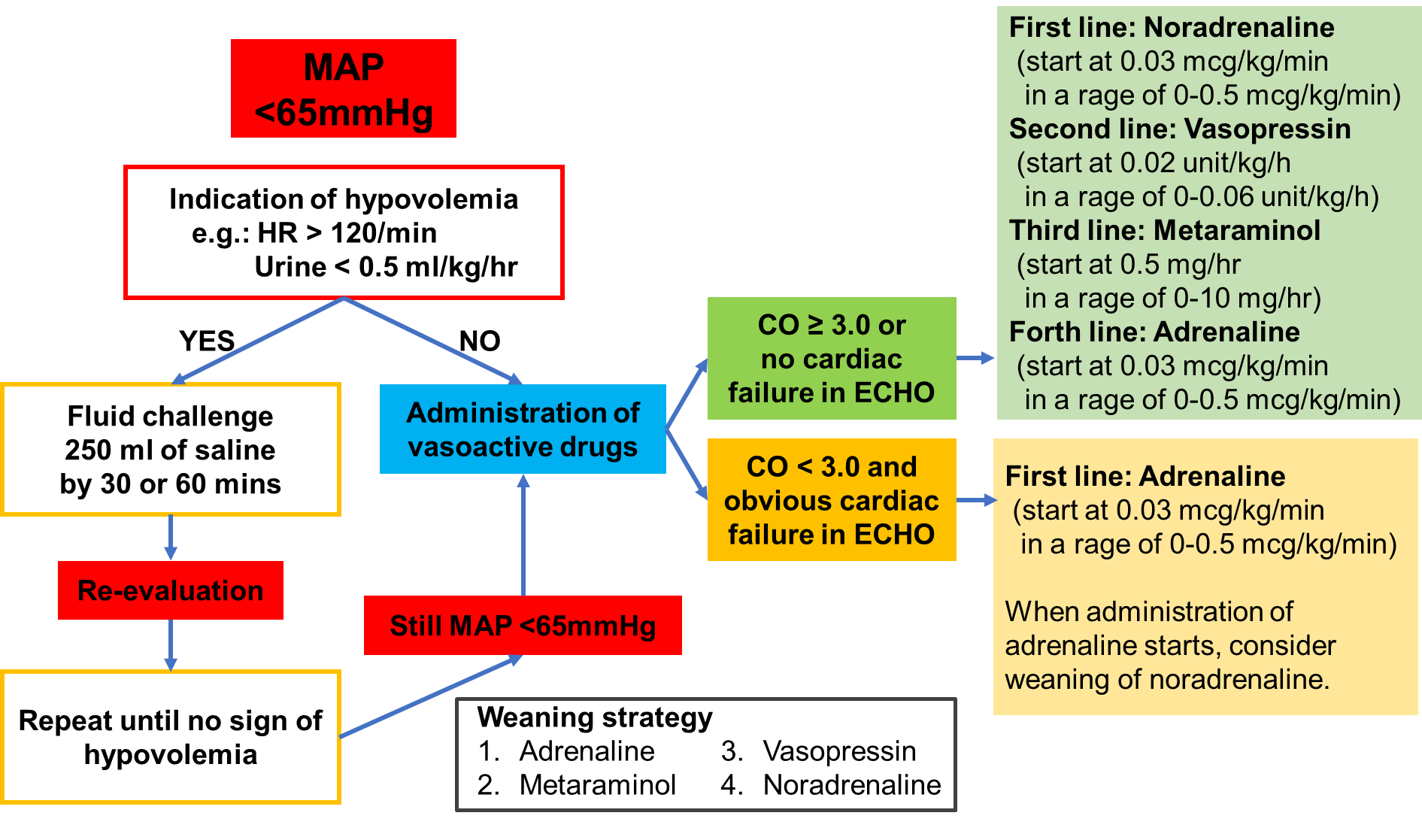


CO: cardiac output, ECHO: echocardiogram (ultrasound image assessment), HR: heart rate, MAP: mean arterial pressure,

**Supplemental Figure 3. Protocol to develop refractory severe ARDS**


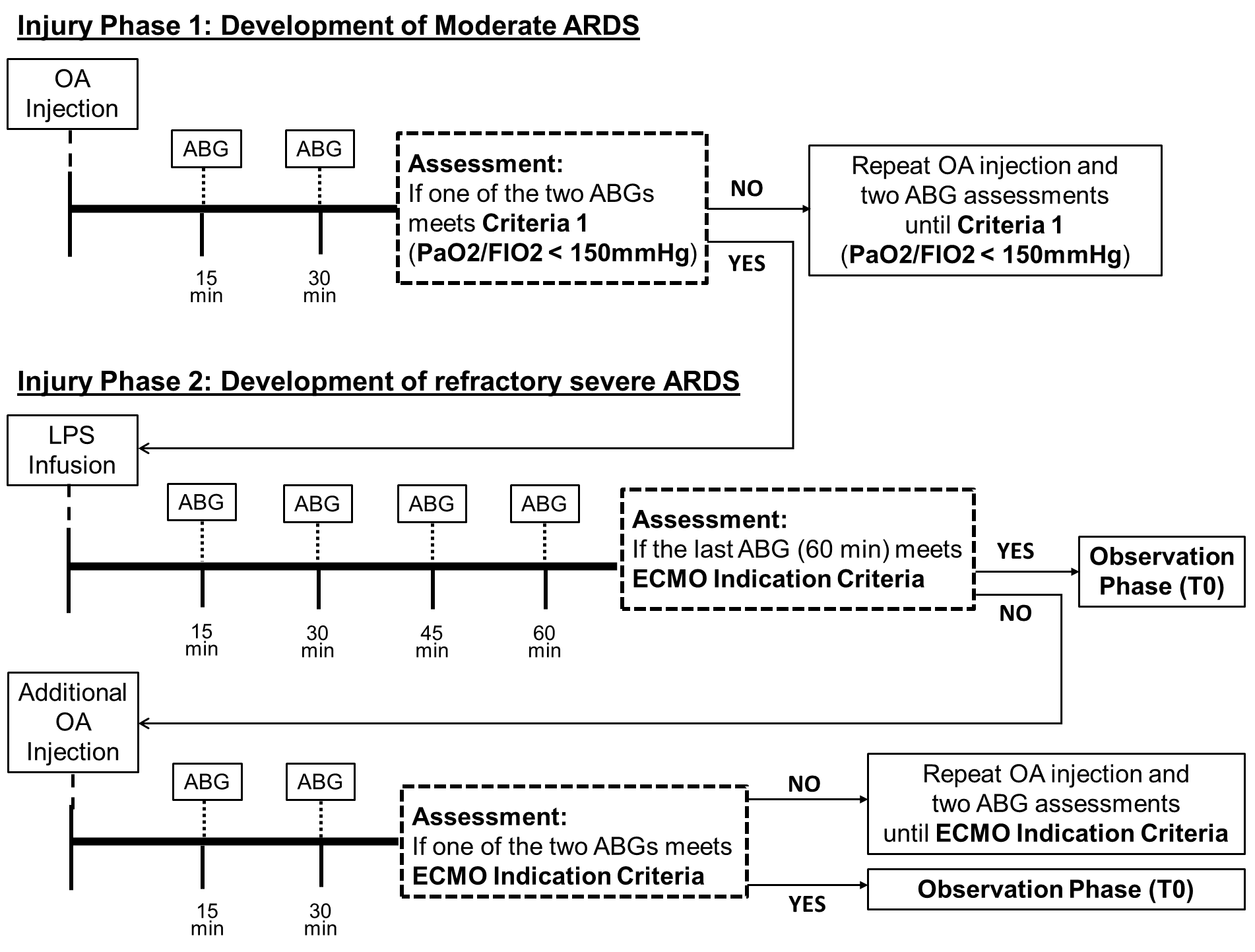


ABG: arterial blood gas, ARDS: acute respiratory distress syndrome, ECMO: extracorporeal membrane oxygenation, FIO_2_: fraction of inspired oxygen, LPS: lipopolysaccharide, OA: oleic acid, PaCO_2_: partial pressure of carbon dioxide, PaO_2_: partial pressure of oxygen, T0: 0 hours after ECMO Indication Criteria

**Supplemental Figure 4. ECMO Indication Criteria**


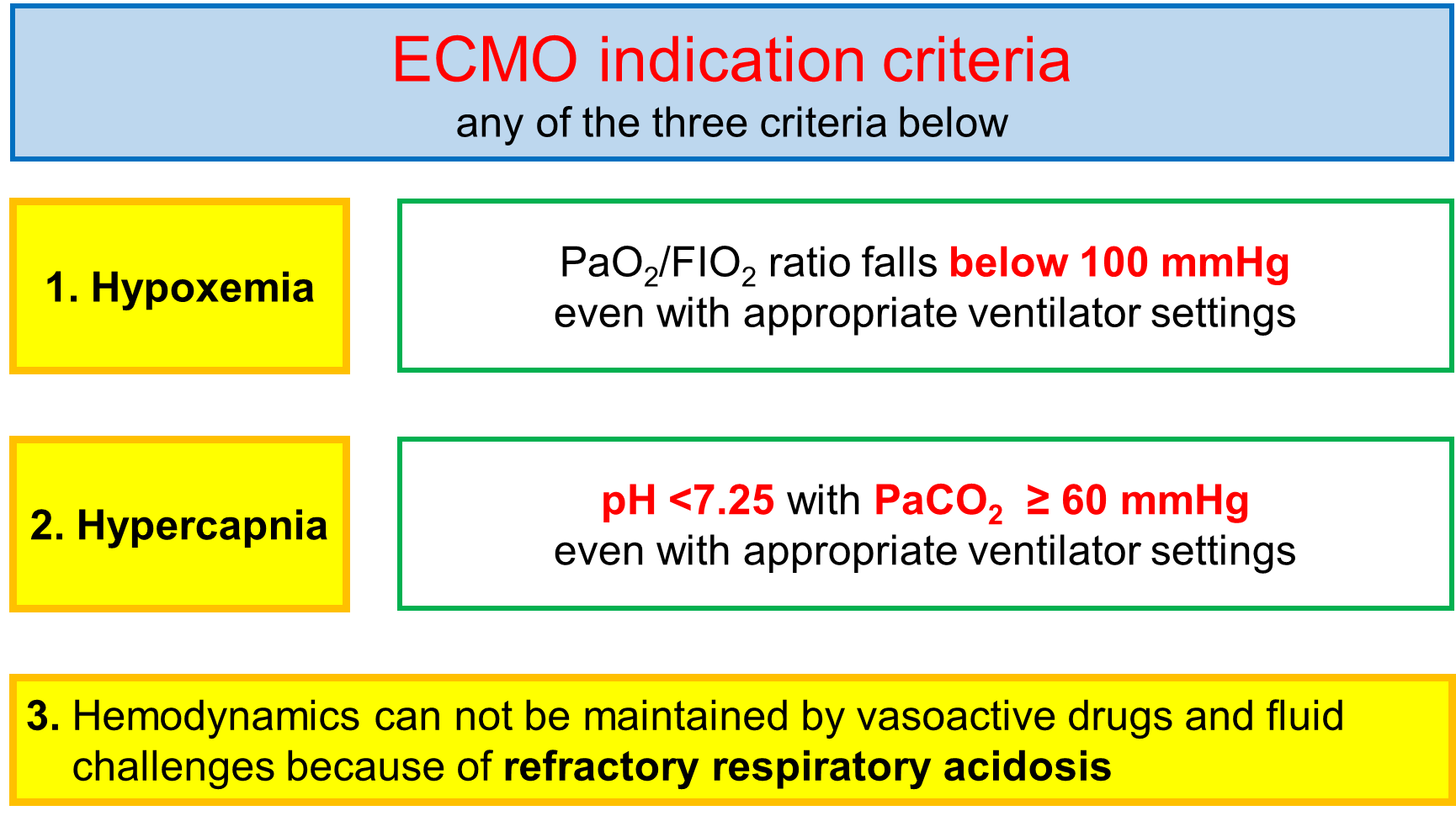


ECMO: extracorporeal membrane oxygenation, FIO_2_: fraction of inspired oxygen, PaCO2: partial pressure of carbon dioxide, PaO2: partial pressure of oxygen

**Supplemental Figure 5. ECMO management protocol**


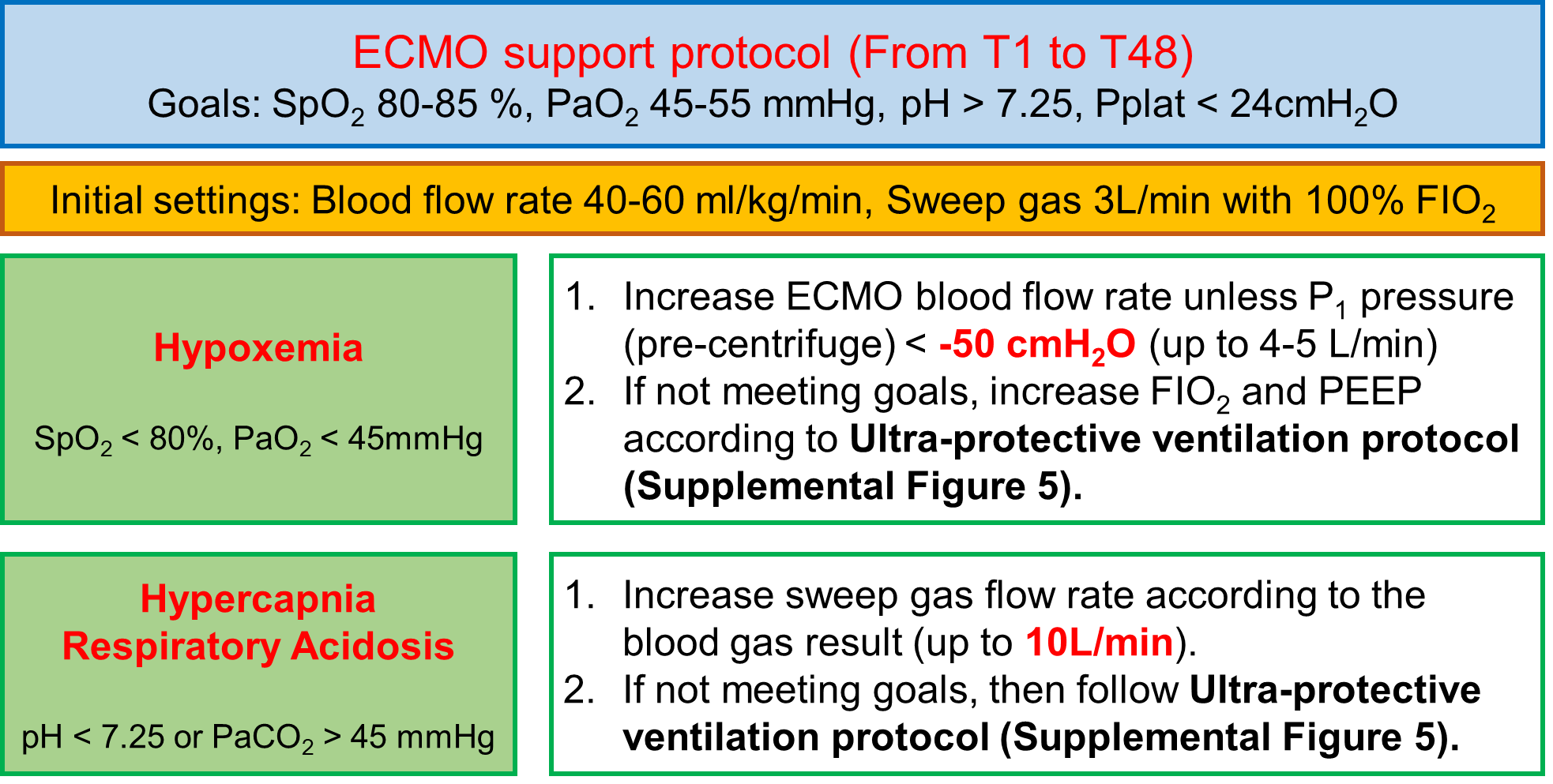


ECMO: extracorporeal membrane oxygenation, FIO_2_: fraction of inspired oxygen, PaCO_2_: partial pressure of carbon dioxide, PaO_2_: partial pressure of oxygen, PEEP: positive end-expiratory pressure, Pplat: plateau pressure, SpO_2_: percutaneous oxygen saturation, TV: tidal volume,

**Supplemental Figure 6. Ultra-protective mechanical ventilation protocol**


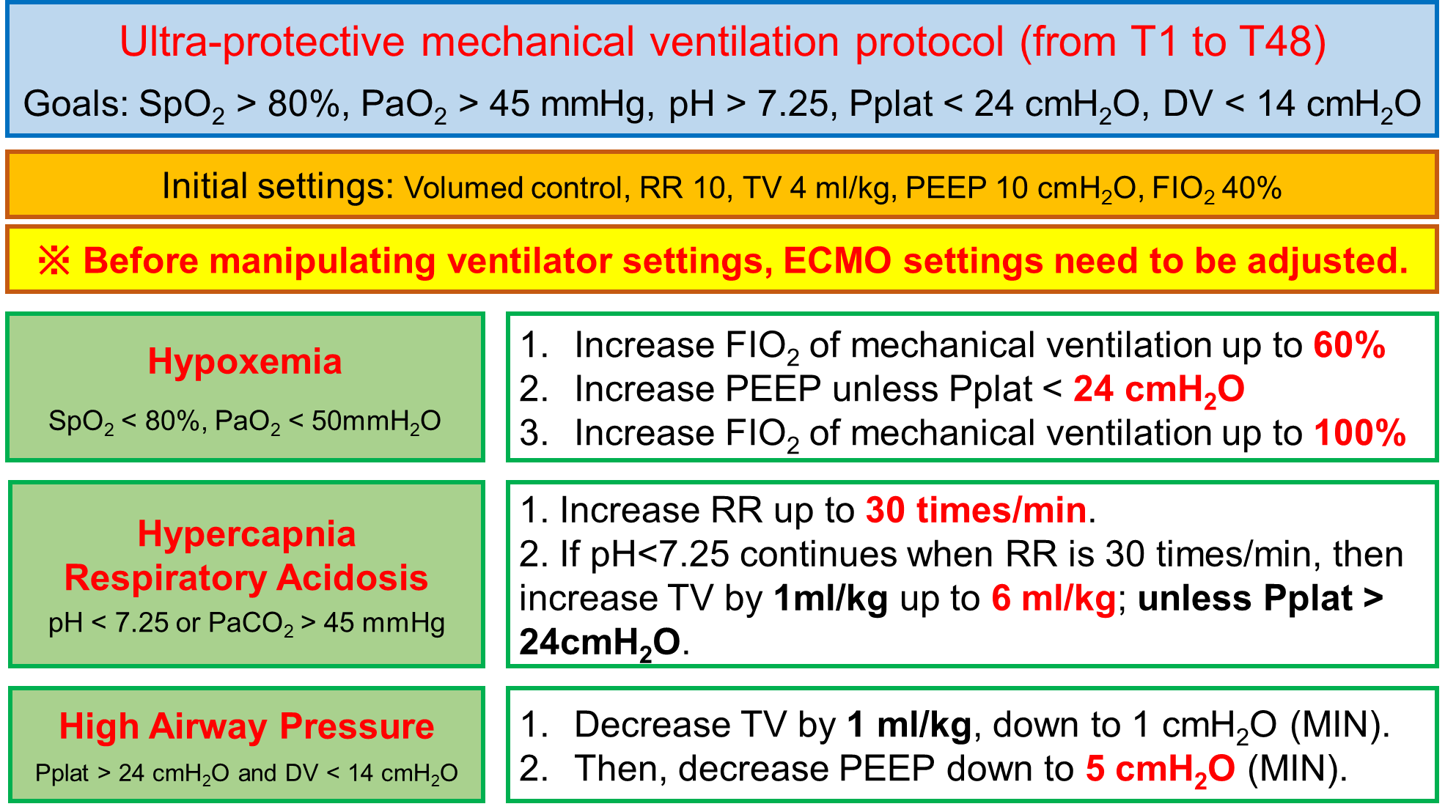


DV: driving pressure (plateau pressure – PEEP), ECMO: extracorporeal membrane oxygenation, FIO_2_: fraction of inspired oxygen, MIN: minimum, PaCO_2_: partial pressure of carbon dioxide, PaO_2_: partial pressure of oxygen, PEEP: positive end-expiratory pressure, Pplat: plateau pressure, RR: respiratory rate, SpO_2_: percutaneous oxygen saturation, TV: tidal volume,

**Supplemental Figure 7. The sampling time of the experiment**


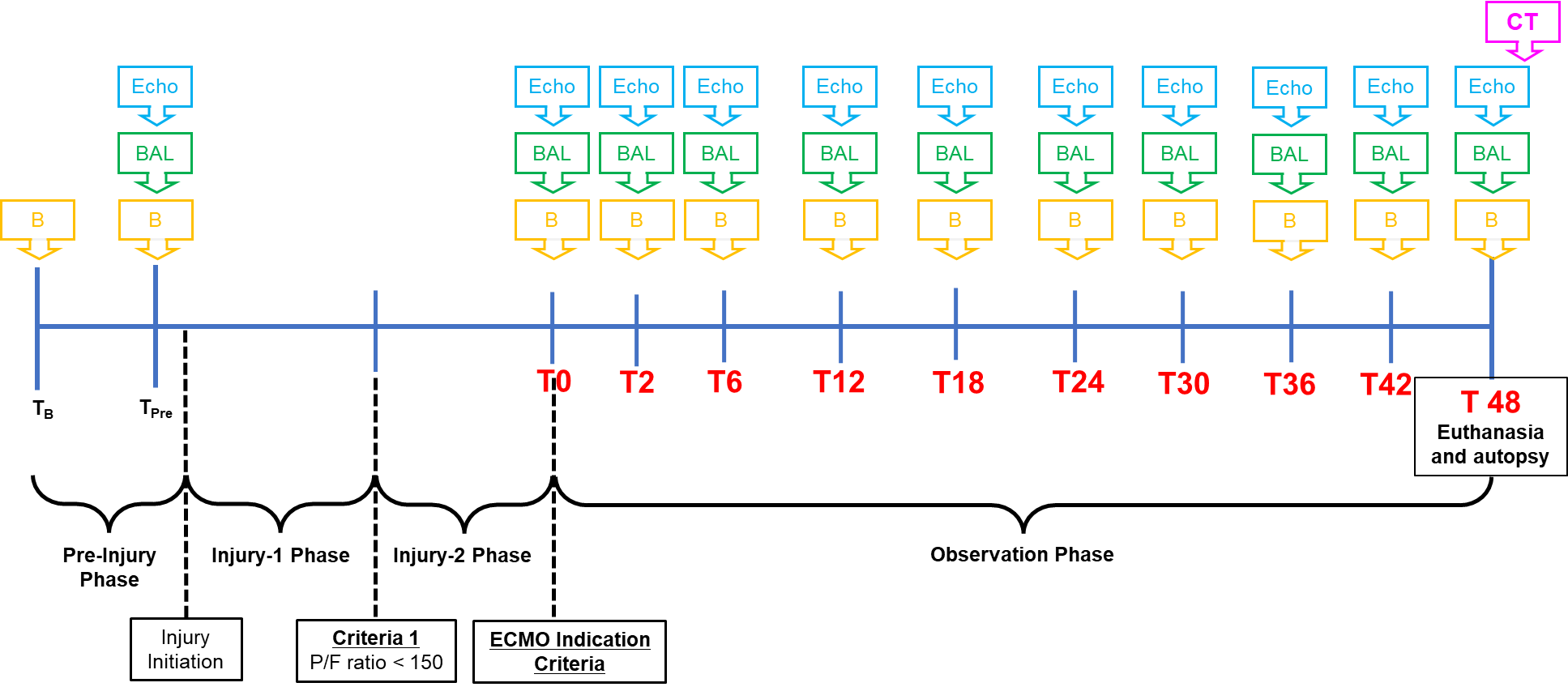


B: blood, BAL: bronchoalveolar lavage, Echo: cardiac and lung ultrasound, CT: computed tomography

**Supplemental Figure 8. Comparison of lung wet-to-dry ratio**


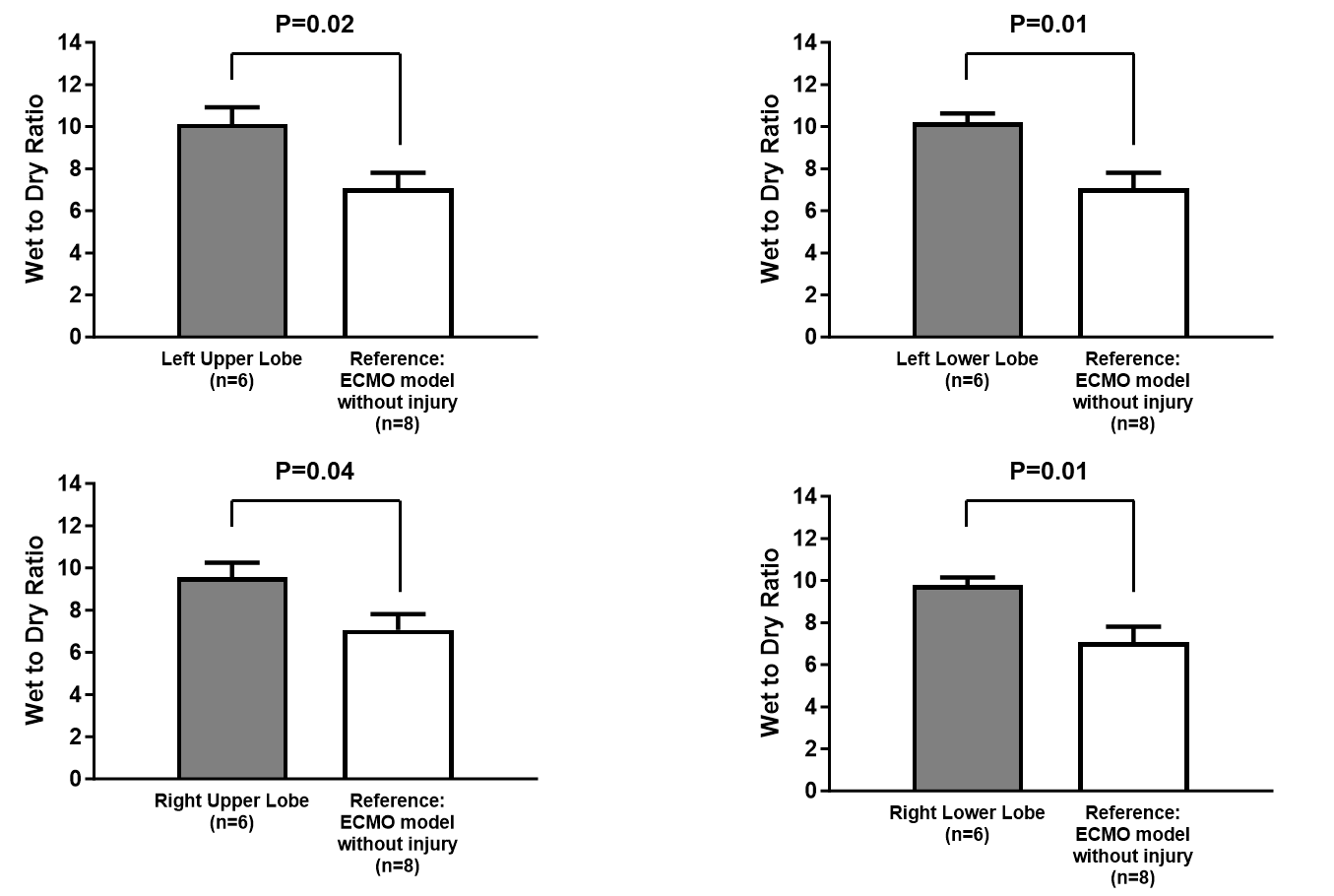


ECMO: extracorporeal membrane oxygenation

The reference of an ovine model of ECMO without injury was published in the previous paper: Passmore MR, Fung YL, Simonova G, et al. Inflammation and lung injury in an ovine model of extracorporeal membrane oxygenation support. Am J Physiol Lung Cell Mol Physiol. 2016;311(6): L1202-L1212. Briefly, the ECMO model was a sham non-injury sheep placed on ECMO lasting for 24 hours while receiving conventional ventilation. Fredman’s test was used for non-parametric analyses. The corrections for multiple testing was not applied.
